# The RNA Export Factor, Nxt1, Is Required for Tissue Specific Transcriptional Regulation

**DOI:** 10.1371/journal.pgen.1003526

**Published:** 2013-06-06

**Authors:** Simona Caporilli, Yachuan Yu, Jianqiao Jiang, Helen White-Cooper

**Affiliations:** School of Biosciences, Cardiff University, Cardiff, United Kingdom; The University of North Carolina at Chapel Hill, United States of America

## Abstract

The highly conserved, Nxf/Nxt (TAP/p15) RNA nuclear export pathway is important for export of most mRNAs from the nucleus, by interacting with mRNAs and promoting their passage through nuclear pores. *Nxt1* is essential for viability; using a partial loss of function allele, we reveal a role for this gene in tissue specific transcription. We show that many *Drosophila melanogaster* testis-specific mRNAs require *Nxt1* for their accumulation. The transcripts that require *Nxt1* also depend on a testis-specific transcription complex, tMAC. We show that loss of *Nxt1* leads to reduced transcription of tMAC targets. A reporter transcript from a tMAC-dependent promoter is under-expressed in *Nxt1* mutants, however the same transcript accumulates in mutants if driven by a tMAC-independent promoter. Thus, in *Drosophila* primary spermatocytes, the transcription factor used to activate expression of a transcript, rather than the RNA sequence itself or the core transcription machinery, determines whether this expression requires *Nxt1*. We additionally find that transcripts from intron-less genes are more sensitive to loss of *Nxt1* function than those from intron-containing genes and propose a mechanism in which transcript processing feeds back to increase activity of a tissue specific transcription complex.

## Author Summary

In multicellular organisms, differentiated cells have a cell-type specific profile of gene expression. Sperm production is particularly specialised, and as a result over 5% of all genes are expressed exclusively in the sperm precursor cells, termed primary spermatocytes. Expression of these genes depends on a particular transcription regulation complex (tMAC) only active in spermatocytes. In this paper we show that a factor, Nxt1, whose previously characterised function is in transport of RNA from the cell nucleus to the cytoplasm, is also required for expression of many testis-specific transcripts. Spermatocytes deficient for Nxt1 fail to express many tMAC-dependent genes, and we show that this effect is due in part to reduced transcription. We further show that processing of the RNA, via splicing, can partially offset the need for Nxt1 in expression of tMAC-dependent genes. Our data reveal an unexpected link between the core RNA processing pathway and a tissue-specific transcription factor.

## Introduction

Export of mRNAs from the nucleus is a fundamental aspect of gene expression regulation. This involves RNA processing and packaging followed by translocation via nuclear pores (reviewed in [1], [2]). During transcription, nascent RNA associates with hnRNPs and a succession of export factors. The THO complex binds transcripts as they emerge from the RNA polymerase, and promotes transcription elongation and 3′ end processing [3]. Associated with THO are UAP56 (an RNA helicase) and REF (an adaptor protein), to make a super-complex, TREX [4]. Splicing occurs co-transcriptionally, and intron excision leads to deposition of the exon junction complex (EJC) 5′ of the splice site [5]. The EJC is also implicated in recruitment of TREX to the nascent transcript [6]. After this initial processing, a dimer comprising Nxf1 (TAP) and Nxt1 (p15) is recruited via interaction of Nxf1 with REF [7]. Recruitment of these export factors renders the mature poly adenylated mRNA competent for export via nuclear pores. mRNAs derived from intronless genes also interact with REF and UAP56, and this again recruits Nxf1/Nxt1 [8]. The Nxf1/Nxt1 dimer is released from the mRNA in the cytoplasm, and recycled to the nucleus. Most RNA polymerase II transcripts are exported via this Nxf1/Nxt1-dependent route, however some endogenous mRNAs and viral RNAs use a parallel route, dependent on the nuclear protein export factor Crm1, acting via unknown RNA-binding adapters [9]. The components of the canonical pathway are well conserved throughout eukaryotes. REF (RNA export factor) is also known as ALY or Aly (ally of AML1 and LEF1), however the protein product of the *Drosophila melanogaster* meiotic arrest transcriptional regulator *aly* (*always early*) is also abbreviated Aly. To avoid confusion, throughout this paper we will use REF to denote the RNA export factor, encoded in *Drosophila* by *Ref1* and *ref2*, and *aly* (Aly) to refer to the *always early* gene (and protein).

Differential gene expression underlies the morphological diversity of differentiated cell types, and depends on regulation at both transcriptional and post-transcriptional levels. The production of mature sperm demonstrates the extreme morphological specialisation achievable, and relies on testis-specific expression of many genes. About 8% of annotated *Drosophila* protein-coding genes are expressed exclusively in testes [10], [11]. Transcription of these genes occurs predominantly in primary spermatocytes [12], [13]. The mRNAs are exported from the nucleus and either translated immediately, or, more typically, are stored in translationally inactive RNPs for translation during spermiogenesis (reviewed in [14]). Most of the “meiotic arrest” genes promote the spermatocyte-specific transcriptional programme (reviewed in [15]). Meiotic arrest testes contain only stages up to and including mature primary spermatocytes and the spermatocytes of almost all meiotic arrest mutants fail to express a very large subset of the testis-specific transcripts, as well as some ubiquitous transcripts [16], [17]. Most meiotic arrest genes encode proteins predicted to form two distinct complexes, tMAC and a testis-specific version of TF_II_D (reviewed in [15]). These complexes are believed to primarily regulate transcription within primary spermatocytes, rather than acting post-transcriptionally. tMAC comprises at least 6 subunits that co-purified from testis extract, encoded by *aly*, *comr*, *tomb*, *topi*, *mip40* and *Caf1* [18]. Genetic and protein interaction data indicate that the complex also probably contains Wuc and Achi/Vis [16], [19]. At least four tMAC subunits (Comr, Tomb, Topi and Achi+Vis) contain predicted DNA-binding motifs, and the complex localises to chromatin in primary spermatocytes, consistent with a transcriptional transactivator role [20], [21], [22], [23], [24]. The testis-specific TATA-binding proteins (tTAFs), subunits of a putative testis form of TF_II_D, and encoded by *can*, *mia*, *nht*, *rye* and *sa*, localise to promoters of target genes and the nucleolus in primary spermatocytes [25], [26], [27]. The meiotic arrest mutants are classified according to phenotype. *aly*-class mutants (*aly*, *comr*, *tomb*, *topi* and *achi+vis*) have virtually undetectable levels of a very large number of transcripts in mutant testes. All *aly*-class mutants encode tMAC subunits. *can*-class mutants (encoded by tTAF genes) have dramatically reduced expression of a large subset of the genes affected in *aly*-class mutants, although they express normal levels of some *aly-*class dependent transcripts [15]. Interestingly, *wuc;aly* double mutant testes, despite being mutant for two tMAC subunits actually has a *can*-class phenotype with respect to defects in gene expression [16]. Recently a new meiotic arrest mutant, *thoc5*, which encodes a THO complex subunit was reported [28]. No transcriptional defects were reported in this mutant, although transcription of only a few genes was assayed.

Here we identify *Nxt1* as a meiotic arrest gene in *Drosophila*, and show that it is a founding member of a new meiotic arrest gene class. Partial loss of function of *Nxt1* results in failure of primary spermatocytes to accumulate many transcripts. Transcripts that were sensitive to loss of *Nxt1* were also dependent on tMAC for their transcription, indicating a mechanistic link between the testis-specific transcription machinery and the core mRNA export pathway. We demonstrate that the link depends on the promoter used to generate the mRNA, and not on the mRNA sequence itself. We found that spliced transcripts are expressed more efficiently in the Nxt1 loss of function background than unspliced transcripts, and propose a model in which RNA processing feeds back to increase the activity of the tissue-specific transcription complex.

## Results

### *Nxt1* is a novel meiotic arrest gene

**Figure 1:**
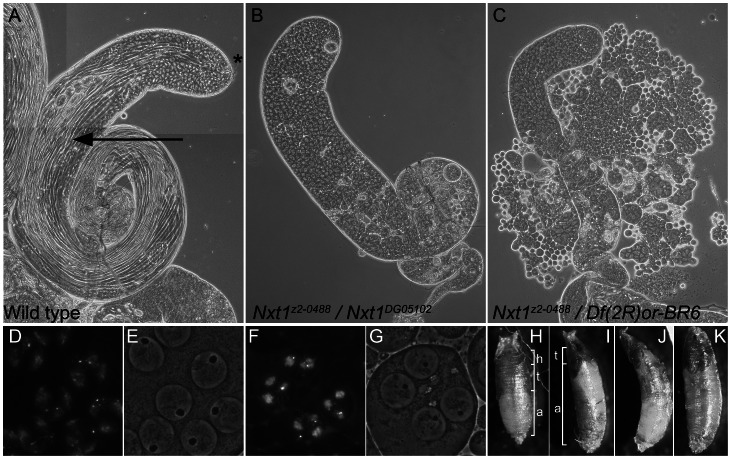
Mutation of *Nxt1* leads to meiotic arrest in testes and failure of head eversion in pupae. (A) All stages of spermatogenesis are seen in wild type testes by phase contrast microscopy. Large round cells near the apical region (*) are primary spermatocytes, while spermatids elongated up the length of the testis (arrow). In *Nxt1^z2-0488^*/*Nxt1^DG05102^* mutant (B) or *Nxt1^z2-0488^*/*Deficiency* (C) testes, only stages up to mature primary spermatocytes are present. (D) Hoechst fluorescence and (E) phase contrast images of wild type mature primary spermatocytes reveals a prominent nucleolus and distinct chromosome territories in each nucleus. (F) Hoechst fluorescence and (G) phase contrast imaging of *Nxt1* mutant spermatocytes reveals that the cells arrest with partially condensed chromatin and a prominent nucleolus. (H) *Nxt1*/+ pupae had everted spiracles, and distinct head, thorax and abdomen (h, t, a), while *Nxt1* mutant pupae (I–K) often had only partially everted spiracles, and the thorax was at the extreme anterior of the pupal case. Many mutant pupae were also curved (J).

**Figure 2:**
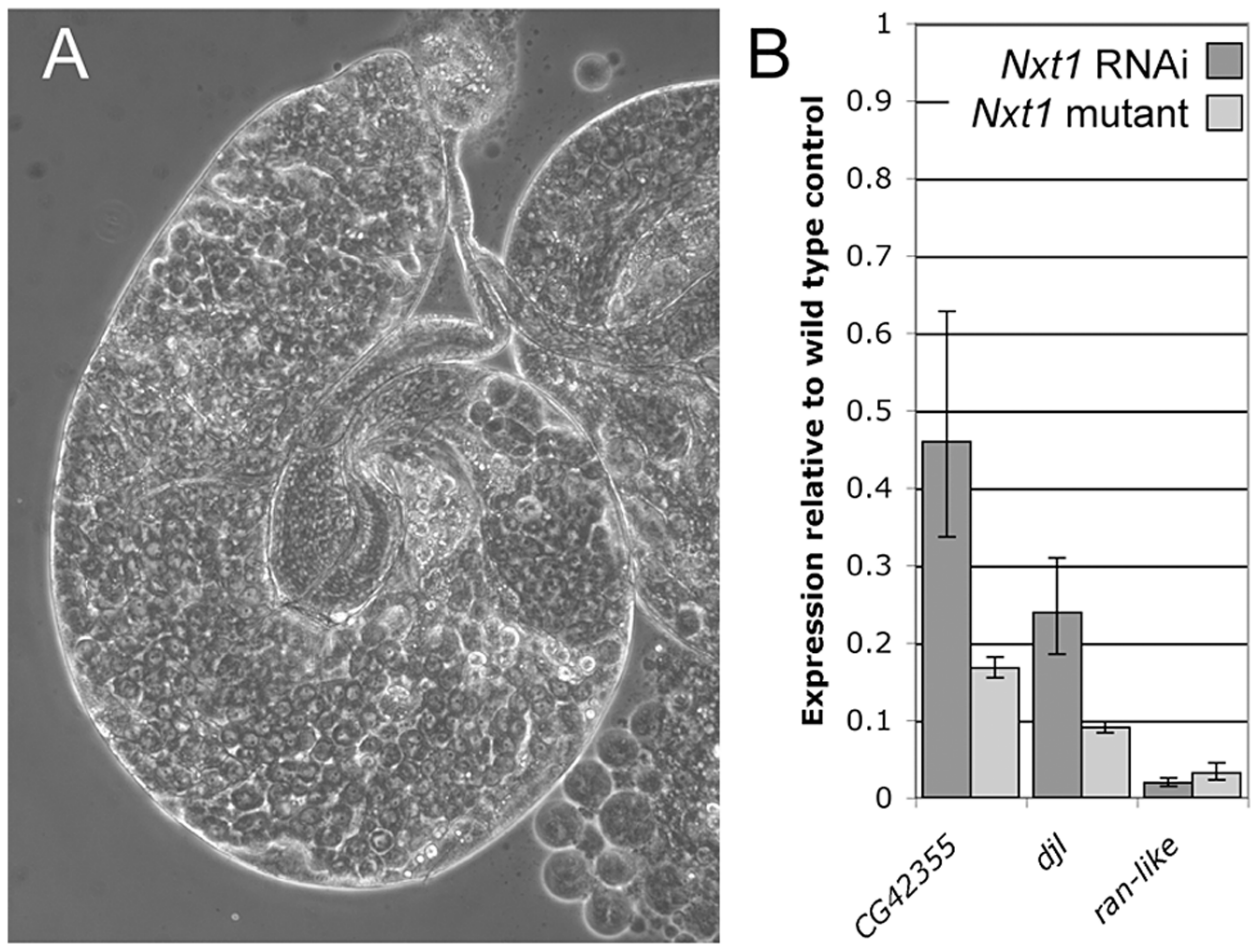
RNAi of *Nxt1* in spermatocytes phenocopies the *Nxt1^z2-0488^* defect. (A) Expression of an *Nxt1* RNAi hairpin construct in the male germline causes a highly penetrant meiotic arrest phenotype. (B) Q-RT-PCR reveals that expression of *Nxt1* target genes is reduced in *Nxt1* RNAi testes (dark bars), and that the effect is similar to that seen in *Nxt1^z2-0488^*/*Nxt1^DG05102^* mutant testes (light bars).

The line *z2-0488* was identified by screening a collection of EMS-induced male sterile mutants using phase-contrast microscopy of live testis squash preparations [29]. *z2-0488* homozygote and hemizygote males showed a typical meiotic arrest phenotype; the mutant testes had stages up to and including mature primary spermatocytes, however the meiotic divisions and spermatid differentiation were absent (Figure 1C). Positional cloning (see Materials and Methods and Figure S1) revealed that *z2-0488* causes an amino acid substitution (D126N) in the RNA export protein Nxt1. An independent allele. *Nxt1^DG05102^*, which has a P-element insertion in the coding region, and is expected to be a null, was obtained from Bloomington *Drosophila* stock centre. *Nxt1^z2-0488^* homozygotes, *Nxt1^z2-0488^* hemizygotes and *Nxt1^z2-0488^*/*Nxt1^DG05102^* transheterozygotes were semi-lethal and male and female sterile. Most of these pupae had head eversion defects during the pupal stages (Figure 1 H–K), accounting for the semi-lethality. Testes from *Nxt1^z2-0488^*/*Nxt1^DG05102^* displayed a meiotic arrest phenotype (Figure 1B). *Nxt1^DG05102^* homozygotes and hemizygotes were embryonic lethal. Depletion of *Nxt1* from spermatocytes by RNAi also generated a meiotic arrest phenotype (Figure 2A), indicating that *Nxt1* functions autonomously in these cells.

**Figure 3:**
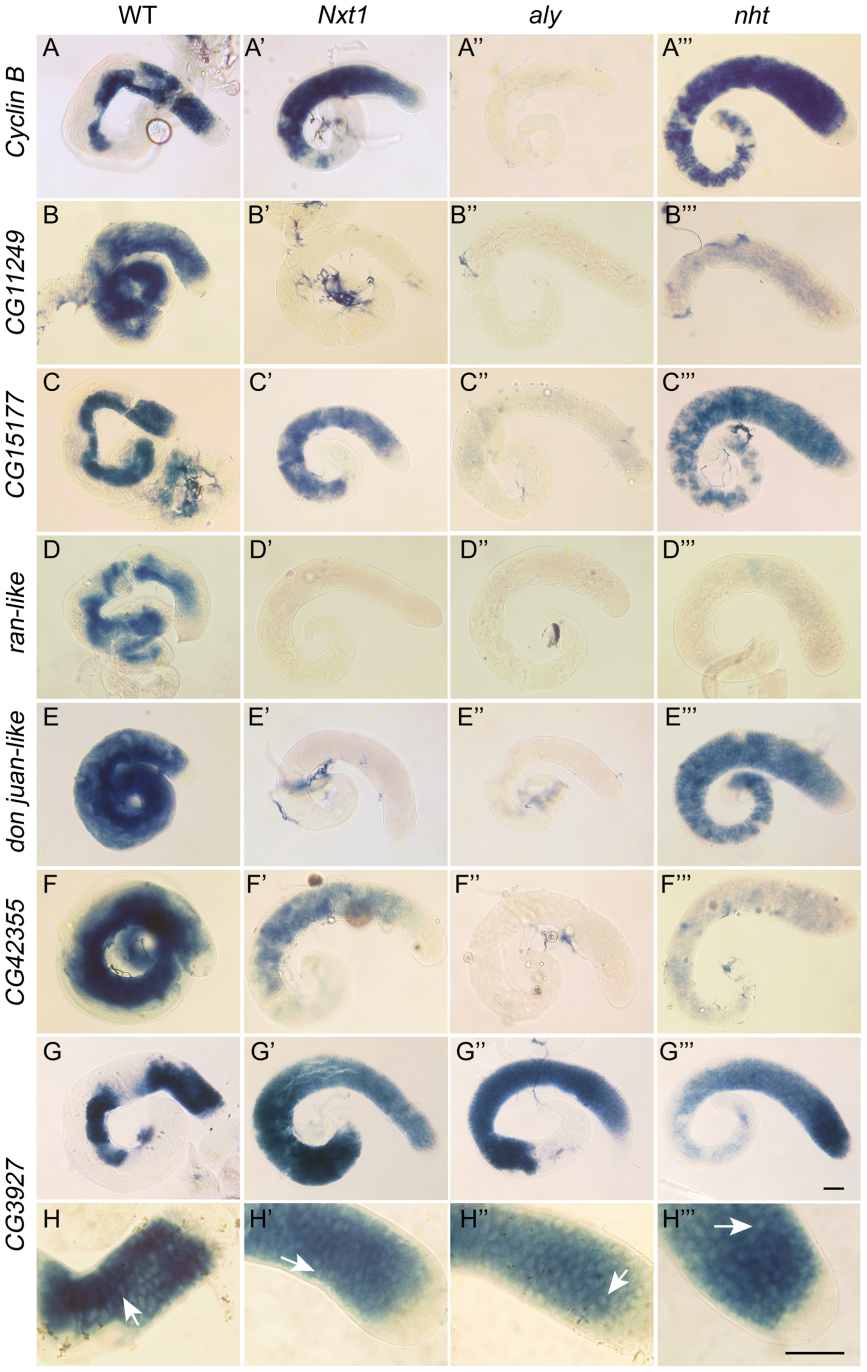
Gene expression defects in *Nxt1* mutant testes places *Nxt1* in a novel meiotic arrest class. Comparison of gene expression defects in Nxt1, *aly* (*aly*-class mutant) and *nht* (*can*-class mutant) by RNA in situ hybridisation. All test genes were expressed in primary spermatocytes of control testes (A–F), with some transcripts persisting into spermatid elongation stages. No signal was detected in tMAC mutant testes (*aly*) (A″–F″), while all were detected at basal level (*CG11249* and *ran-like*) or higher (*CycB*, *CG15177*, *djl*) in *nht* testes (A′″–F′″). *CycB*, *CG15177* and *CG42355* expression was detected in *Nxt1^z2-0488^*/*Nxt1^DG05102^* mutant testes (A′″, C′″, F′″) while *CG11249*, *ran-like* and *djl*, were not detected in *Nxt1* testes. *CG3927* expression (control) is restricted to primary spermatocytes, and robust expression was detected in all four genotypes (G–G′″). Higher magnification images of the CG3927 staining (H–H′″) show a honey-comb appearance of the signal in all four genotypes, indicating mRNA accumulation predominantly in the cytoplasm rather than the nucleus (arrows). Scale bars are 50 µm. Bar in G′″ applies to A–G′″, bar in H′″ applies to H–H′″.

We conducted a detailed phenotypic analysis to compare the meiotic arrest caused by *Nxt1* mutation with previously described meiotic arrest loci. RNA *in situ* hybridisation revealed that *Nxt1* mutant testes had defects in accumulation of some tMAC target mRNAs, while other tMAC target transcripts were detected in the cytoplasm of the mutant primary spermatocytes (Figure 3). *Nxt1* mutant testes failed to express some genes that were expressed in *nht*, a *can*-class mutant (Figure 3). The RNA *in situ* hybridisation profiles thus did not match those seen in any characterised class of meiotic arrest mutant (Figure 3), suggesting that *Nxt1* is the founding member of a novel class. Surprisingly, given the known role of Nxt1 in mRNA export from the nucleus [30], we detected no accumulation of transcripts within the nucleoplasm. All mRNAs detected were exclusively cytoplasmic (Figure 3H–H′″).

Uniquely among meiotic arrest mutants, *thoc5* primary spermatocytes showed severe fragmentation and disorganisation of the nucleolus [28]. Phase contrast microscopy revealed no overt nucleolar organisation defect in *Nxt1* mutant testes, although the nucleoli in some mature primary spermatocytes appeared smaller than in wild type (Figure 1D–G). Sa-GFP, whose localisation is abnormal in both *aly*-class and *thoc5* mutant testes, was localised to the nucleolus and indistinguishable from wild type in *Nxt1* primary spermatocyte (data not shown). The chromatin in arrested spermatocytes was partially condensed, and displaced from the nuclear envelope (Figure 1F, G).

### The subcellular localisation of Nxt1 is dynamic in male germline cells

**Figure 4:**
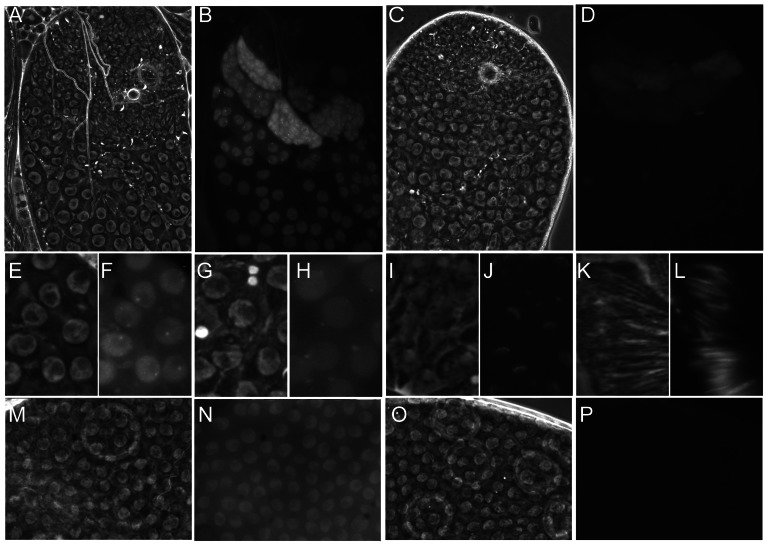
The dynamic localisation of eGFP-Nxt1. Phase contrast (A, C, E, G, I, K, M, O) and fluorescence images (B, D, F, H, J, L, N, P) of ectopically expressed eGFP-Nxt1(wild type) or eGFP-Nxt1-D126N (D, P). (A–D) Testes tips oriented with younger cells towards the top. When expressed in male germline cells the wild type protein (A, B) localised predominantly to the nucleus, and remained stable as spermatocytes matured. The signal from mutant protein was dramatically weaker, and predominantly cytoplasmic (C, D). In early primary spermatocytes the WT protein localised to an intra-nuclear dot, adjacent to the nucleolus (F) as well as throughout the nucleoplasm. As spermatocytes matured the protein relocated to one or more cytoplasmic puncta, frequently found adjacent to the uniformly labelled nucleus (H). Early elongation spermatids showed cytoplasmic puncta and eGFP-Nxt1 localisation to one face of the nuclear envelope (J). Label persisted until late elongation in spermatid nuclei (L). In the ovarian follicular epithelium the wild type eGFP-tagged protein was predominantly nuclear (N), while the D126N form of the protein was not detected (P).

We determined the sub-cellular localisation of Nxt1 protein by expressing eGFP-Nxt1 using the GAL4-UAS system. When expressed in spermatocytes (with bam-Gal4VP16), the protein was primarily nuclear, although some cytoplasmic signal was detected (Figure 4A, B). Within early primary spermatocyte nuclei the signal was weaker in the nucleolus, and enriched in a peri-nucleolar dot (Figure 4E, F). As primary spermatocytes matured this peri-nucleolar dot disappeared, and one or more cytoplasmic puncta appeared, typically apposed to the nuclear membrane (Figure 4G, H). The protein remained stable into spermatid differentiation, and was localised to one face of the nuclear envelope of early elongation stage spermatids (Figure 4I, J). The protein remained detectable in the needle-shaped nuclei of spermatids (Figure 4K, L), but was not detected in mature sperm. When eGFP-Nxt1 was expressed in ovarian somatic cells using a ubiquitous driver (tubulin-Gal4) it was predominantly nuclear; relatively uniformly distributed within the nucleoplasm, but weaker in the nucleolus. Some cytoplasmic signal was detected (Figure 4M, N).

### Nxt1 protein instability caused by D126N

**Figure 5:**
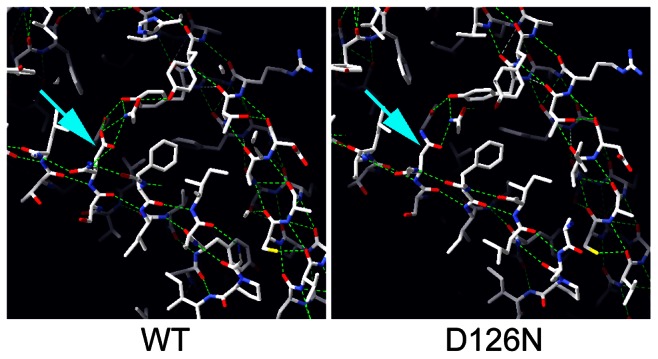
Modelling the potential effect of D126N. The human Nxt1 protein structure was used to model the *Drosophila* Nxt1 protein folding (WT) using Swisspdb viewer, and the region containing the mutated residue is shown. The side chain of D126 is indicated with an arrow. Computed H-bonds are shown in green. Running the model with the D126N substitution (arrow) revealed disruption of two computed H-bonds.

The crystal structure of the human Nxf1/Nxt1 dimer has been solved, and we used this structure to predict the molecular effects of the D126N mutation [31]. The residue is conserved in metazoa (D131 in human Nxt1), and lies on a beta sheet at the Nxt1/Nxf1 dimer interface. The D131 side chain is oriented towards the core of the protein, and participates in a hydrogen bonding network with three other residues, Y24, Y39 and Q111. All three of these residues are conserved throughout metazoa, suggesting that this H-bonding network is conserved. Substitution of D131N disrupts the H-bonding network (Figure 5). This is likely to destabilise the folding around the core of the protein, particularly the interaction of the beta sheets of the dimer interface and an alpha helix on the opposite protein face. Expression of GFP-Nxt1-D126N revealed a dramatic decrease in protein stability (Figure 4). When driven with bam-Gal4VP16, fusion protein was only detected in the cells in which the driver itself was active (Figure 4C, D). The fusion protein in these cells was uniformly distributed. When expressed using tubulin-Gal4 no fusion protein could be detected under conditions in which the wild type protein was detected (Figure 4O, P). The mutation is not predicted to affect the dimer interaction with Nxf1, thus we conclude that the primary defect in the mutant is reduced protein stability, but expect that any mutant protein that does get folded is likely to be able to participate in mRNA nuclear export.

### Many testis-specific transcripts fail to accumulate in *Nxt1* mutant testes

**Figure 6:**
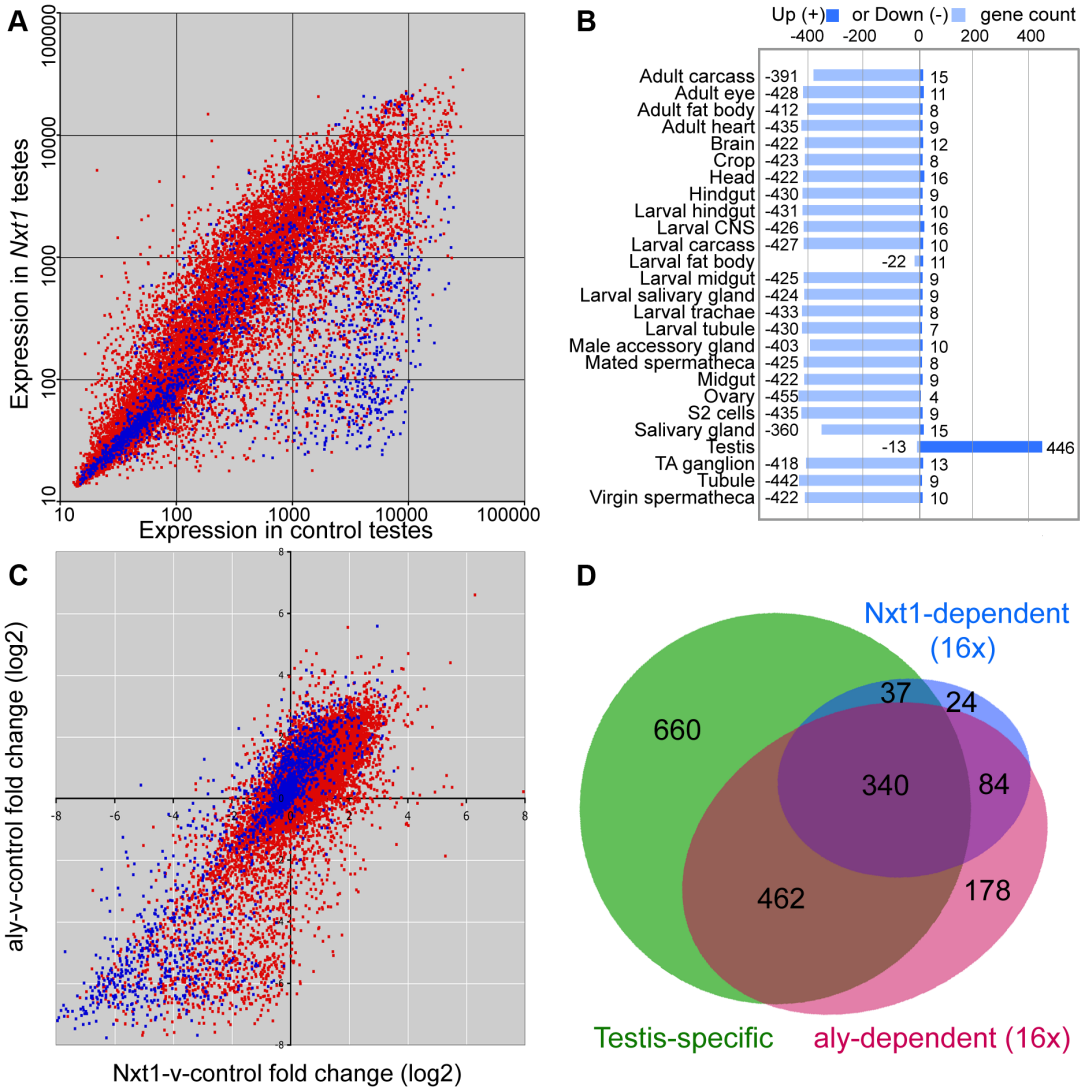
Microarray analysis of *Nxt1*-dependent gene expression in testes. (A) Scatter plot showing normalised expression levels in control testes vs. *Nxt1^z2-0488^*/*Nxt1^DG05102^* mutant testes of all probes on the Affymetrix expression arrays. Red dots correspond to probes for genes with introns, blue dots are probes for intron-less genes. (B) FlyAtlas expression data for 485 probes that are 16× or more down-regulated in *Nxt1* testes compared to control. “Up” indicates probes whose signal is 2× or more higher in the specific tissue than whole fly, “Down” indicates 2× or more lower signal in the specific tissue than whole fly. TA ganglion = Thoracioabominal ganglion. (C) Scatter plots showing log2-transformed fold changes in *Nxt1*-vs-control and *aly*-vs.-control pairwise combination. Red indicates intron-containing genes, blue represents intron-less genes. (D) Venn diagram to show that most highly *Nxt1*-dependent genes (blue) have testis-specific expression (green), and that most highly *Nxt1*-dependent genes are also highly *aly*-dependent (red), but not *vice versa*.

We used microarrays to characterise the gene expression defects in *Nxt1* mutant testes. We found that *Nxt1* mutant testes had dramatic defects in transcript accumulation, with many transcripts being reduced in abundance, or undetectable (Figure 6A). As with other meiotic arrest loci, there were very few transcripts whose level was elevated in mutant testes compared to controls (Figure 6A, Figure S2). 485 probes showed a 16 fold or more decrease in signal in *Nxt1* mutant testes compared to normal testes. Comparison with FlyAtlas data revealed that the transcripts highly dependent on *Nxt1* for expression in testes were strongly biased towards testis-specific expression (Figure 6B). 78% of all highly *Nxt1*-dependent transcripts were testis-specific (Figure 6D), although clearly the majority of testis-specific transcripts are not highly *Nxt1*-dependent (defined as 16× or more down-regulated in *Nxt1* testes). To test whether the transcription defects were a peculiarity of our EMS allele, we assayed gene expression changes in the meiotic arrest testes generated by *Nxt1* RNAi in spermatocytes. We found a very dramatic down-regulation of *ran-like*, and a mild down-regulation of expression of *djl* and *CG42355*, mirroring the results seen with *Nxt1^z2-0488^*/*Nxt1^DG05102^* (Figure 2B).

We compared the global effects on gene expression of *Nxt1* mutation with microarray data from *aly* testes [16]. 1064 probes were highly (16× or more) down-regulated in *aly* testes. Comparison of the fold-change in *Nxt1* vs. control and *aly* vs. control revealed that 424 (87%) of the 485 probes that were highly dependent on *Nxt1* for expression were also highly dependent on *aly* for expression. (Figure 6C, D; Figure S2). Of the remaining 61 highly *Nxt1-*dependent probes, 34 were 8–16 fold down-regulated in *aly*, 24 were 2–8 fold down-regulated in *aly* and only 3 were normal or nearly-normal (less than 2× down) in *aly*. In contrast, the 1064 highly *aly-*dependent probes showed a graded response to loss of *Nxt1*. 424 (40%) were also highly dependent on *Nxt1* (16 fold change), 186 (17.5%) showed a strong requirement for Nxt1 (8–16 fold change), 313 (29.5%) showed a mild-moderate reduction in expression (2–8 fold change) while 141 (13%) were detected at normal, or near-normal (less than 2 fold change) levels in mutant testes (Figure 6C, D; Figure S2). We did not see any reduction in the levels of transcript for any of the known tMAC subunits in *Nxt1* testes (Figure S2). Immunohistochemistry indicated that Aly protein is expressed and localised to the nucleus as per wild type in *Nxt1* testes (data not shown). The finding that many highly *aly*-dependent transcripts are expressed at near-normal levels in *Nxt1* testes, coupled with the observation that no known tMAC components are under-expressed, indicate that the transcript accumulation defects seen in *Nxt1* testes are very unlikely to be caused indirectly, via reduction in expression of a tMAC subunit. These data confirm that *Nxt1* founds a novel meiotic arrest class of mutant, and is required for accumulation in testes of a large set of predominantly testis-specific transcripts that depend on tMAC for transcription.

### The failure to accumulate target mRNAs in *Nxt1* testes is primarily due to reduced transcription

**Figure 7:**
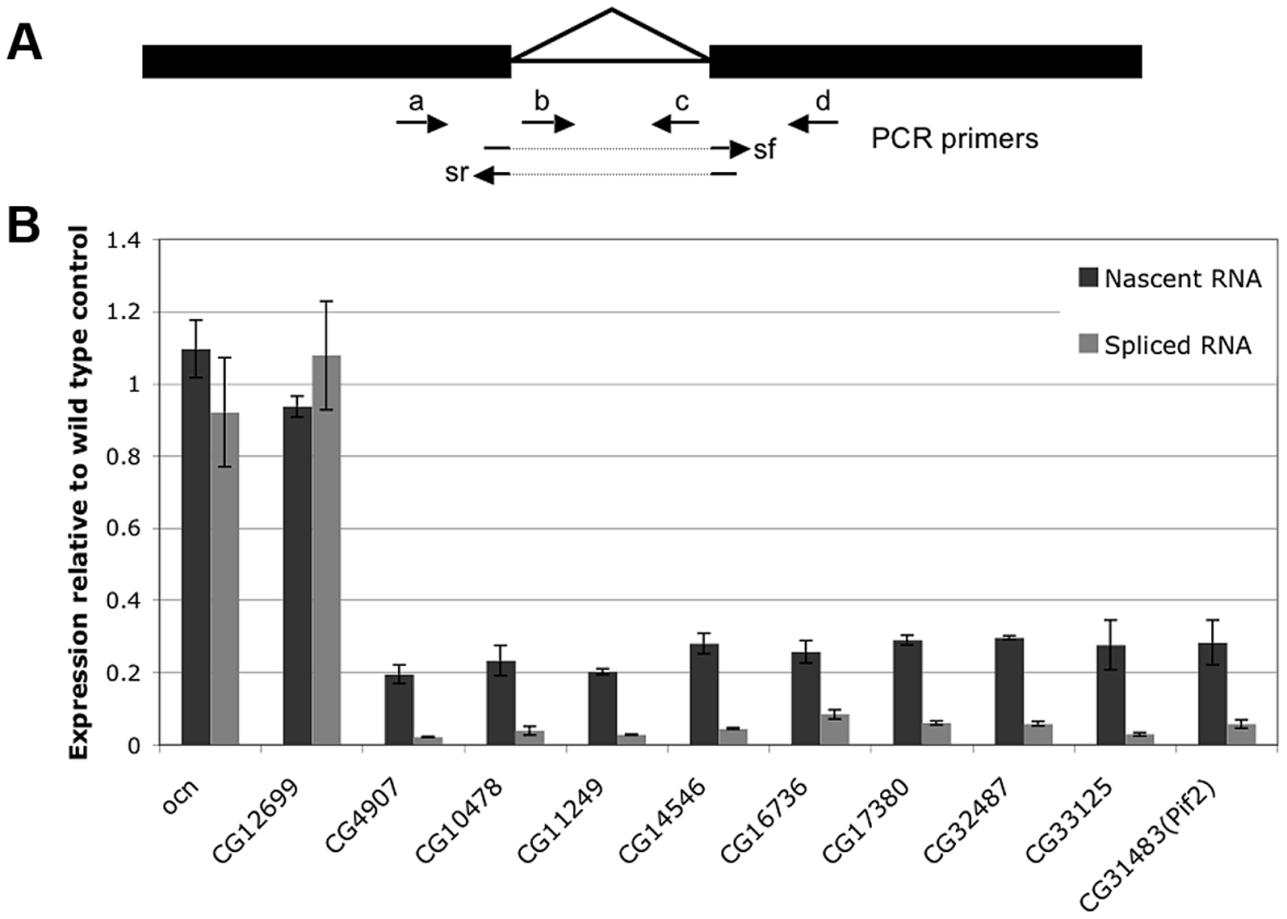
Expression of both nascent and mature mRNA of target genes is reduced in *Nxt1* mutant spermatocytes. (A) Expression of nascent (unspliced) RNA was quantified using primer pairs a+c, b+c or b+d, while mRNA levels were determined with primer pairs a+sr or sf+d. (B) *CG12699* and *ocn* control genes were expressed at broadly similar levels in WT and mutant testes, while expression of both nascent and mature RNA for the Nxt1-dependent test genes *CG4907*, *CG10478*, *CG11249*, *CG14546*, *CG16736*, *CG17380*, *CG32487*, *CG33125* and *pif2* were reduced. For each gene, the expression levels of nascent transcript (dark gray) and mature transcript (light gray) in *Nxt1^z2-0488^*/*Nxt1^DG05102^* are presented as relative levels to those found in the wild-type (arbitrarily assigned as 1).

In light of the known role of *Nxt1* in RNA nuclear export we tested whether the gene expression defects in *Nxt1* testes are the consequence of reduced transcription, or whether normal transcription occurs but the transcripts have reduced stability, or both. We compared the levels of nascent RNA to mRNA in wild type and mutant cells. If transcripts are produced in the mutant, and then degraded, the level of nascent RNA would be similar to WT, while the mRNA level is reduced. If transcription itself is reduced in the mutant then both RNA species would be lower than in WT. To distinguish between nascent and mature RNA we generated cDNA from DNase-treated total spermatocyte RNA using random primers. We amplified nascent RNA with at least one primer in an intron; for mRNA we placed one primer in an exon and the other spanning an exon-exon junction (Figure 7A). All genes selected for this analysis had a single, small, intron. Control genes, *ocn* and *CG12699* were selected that showed no defect in expression in *Nxt1* according to the microarrays. mRNAs and nascent RNAs of these control genes were expressed in mutant spermatocytes at levels similar to WT (Figure 7B). 9 genes (*CG4907*, *CG10478*, *CG11249*, CG14546, *CG16736*, *CG17380*, *CG32487*, *CG33125* and *pif2*) were selected as being highly dependent on *Nxt1* from the microarrays. For all these test genes we saw a reduction in the mature transcript level of 20–40 fold. Analysis of the nascent transcripts revealed a reduction of about 5 fold (Figure 7B). Thus the failure of *Nxt1* mutant testes to accumulate testis-specific transcripts can partially be explained by reduced transcription of these genes.

### tMAC imposes a requirement for Nxt1 on the transcripts whose expression it promotes

**Figure 8:**
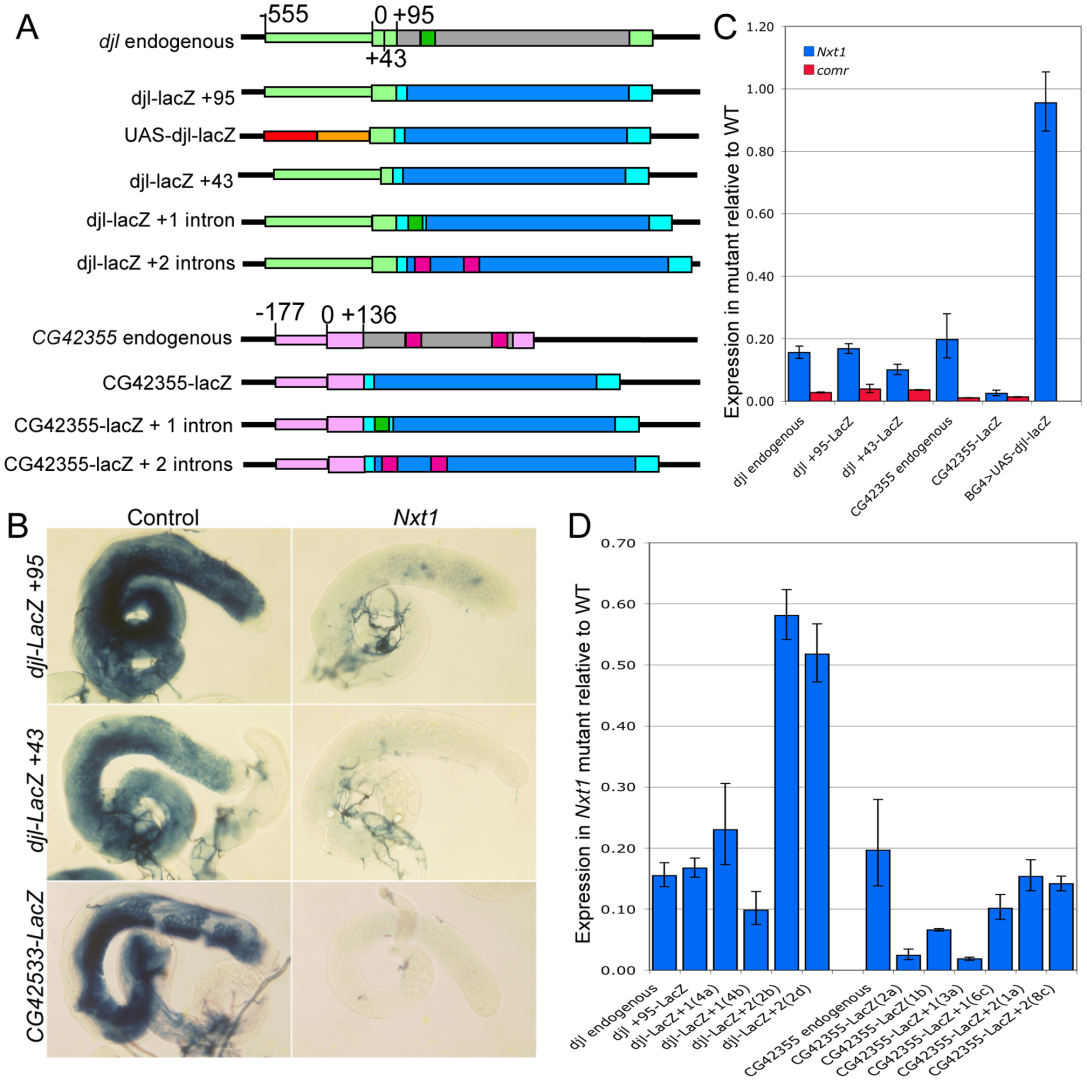
*Nxt1* is required for full expression of tMAC dependent reporter constructs. (A) Schematic diagram to illustrate reporter construct design. Promoter regions are narrow coloured boxes, transcribed regions are broad boxes. The *djl* promoter and UTRs are light green; ORF grey; intron dark green. *CG42355* promoter and UTRs are pale pink; ORF grey; introns dark pink. UAS-djl-LacZ uses 5×UAS (red) and hsp70 minimal promoter to the TSS (orange) as the promoter. All reporter transcripts comprise 5′ UTR (from *djl* or *CG42355*) fused to *Adh* 5′UTR (cyan) LacZ ORF (blue) and SV40 3′UTR (cyan). (B) RNA in situ hybridisation with a *LacZ* probe reveals reduced expression of reporter constructs in *Nxt1^z2-0488^*/*Nxt1^DG05102^* mutant testes compared to control. (C) q-RT-PCR to test *LacZ* reporter expression in mutant spermatocytes compared to controls. Expression for each transgene was normalised as 1 in the control, and the relative expression in *Nxt1^z2-0488^* homozygotes (blue) and *comr* (red) mutants was calculated. (D) q-RT-PCR to test the effect of inclusion of introns on reporter expression in *Nxt1^z2-0488^* homozygote spermatocytes compared to controls. Expression for each transgene was normalised to 1 in the control samples and relative expression in mutant was calculated. Introduction of two introns into the reporters increased expression in the mutant testes compared to the no intron version. Two separate reporter lines for transgenes are shown.

The finding that *Nxt1*-dependent transcripts all rely on tMAC for their expression suggests that the transcriptional regulation could be key to determining whether a specific transcript requires Nxt1 for accumulation. We tested this using reporter constructs in which a testis-specific transcriptional control element is used to drive expression of *LacZ* (Figure 8A). *djl* is highly transcribed in primary spermatocytes, and the transcript remains stable until late elongation, when it is translated. *djl* is testis-specifically expressed and depends strongly on the tMAC component *aly*, but less strongly on *Nxt1*, for its expression (Figure 3, E–E′″, Figure 8C). A genomic fragment spanning the region from −555 to +95 (relative to the transcription start site, TSS) is sufficient to drive transcription of the reporter in primary spermatocytes, and to confer translational repression on the mRNA [32]. This transcription has already been shown to be reduced in tTAF mutants [32]. RNA *in situ* hybridisation (data not shown) and q-RT-PCR (Figure 8C) revealed, as expected, that this *djl+95-LacZ* reporter construct expression is extremely low in a tMAC mutant (*comr*). We found a dramatic reduction in reporter expression in *Nxt1* mutant primary spermatocytes compared to wild type by q-RT-PCR and RNA *in situ* hybridisation (Figure 8B, C). This indicates that the promoter and/or 5′UTR are critical for determining *Nxt1* dependence.

To test whether the *Nxt1*-dependence of a specific transcript depends on the transcript sequence or on the promoter used to activate transcription, we drove expression of the djl+95-LacZ transcript independently of the *djl* promoter by replacing the *djl* promoter with 5×UAS and the *Hsp70* minimal promoter (up to the TSS, Figure 8A). q-RT-PCR of spermatocyte samples revealed that reporter expression in *Nxt1* mutant cells (*w ; Nxt1^z2-0488^*/*Nxt1^DG05102^ ; UAS-djl-LacZ*/*bamGal4VP16*) was essentially equal to that in control cells (*w ; Sco*/*Nxt1^DG05102^ ; UAS-djl-LacZ*/*bamGal4VP16*) when driven using bamGal4VP16 (Figure 8C). The transcripts from djl+95-LacZ and UAS-djl-LacZ have the same sequence; they differ only in the promoter sequence used to activate their expression. Their differential requirement for Nxt1 in accumulation of the transcript therefore reveals that the promoter sequence, rather then the RNA sequence, imposes Nxt1-dependence on the transcript.

**Figure 9:**
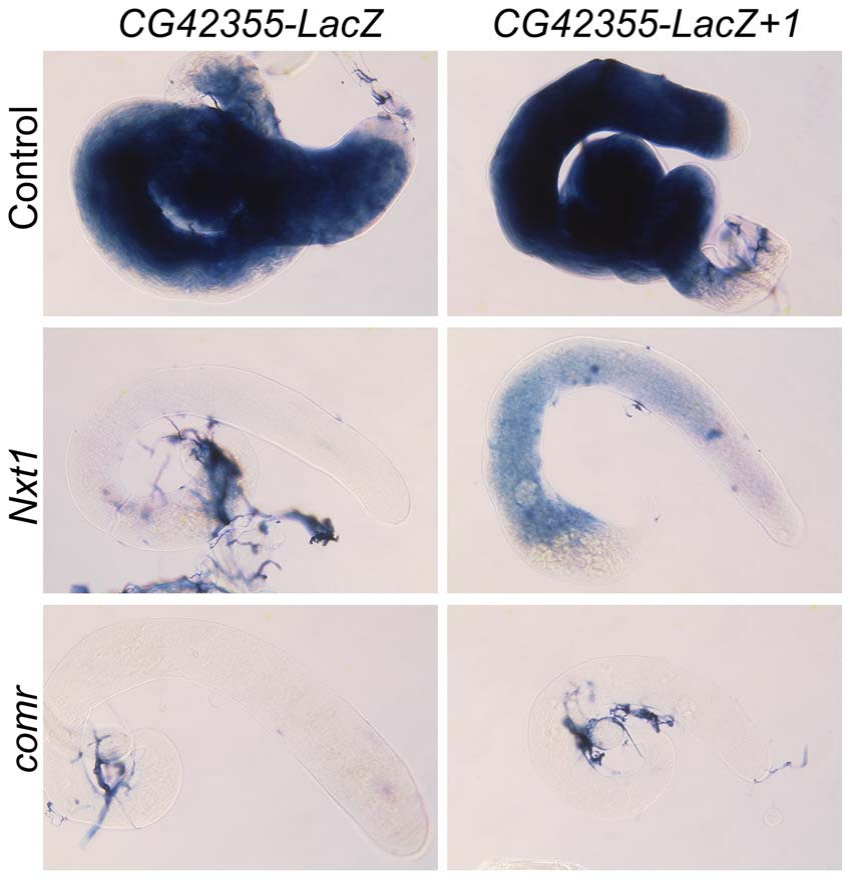
Addition of introns partially restores reporter expression in *Nxt1* but not *comr*. RNA in situ hybridisation reveals CG42355-LacZ reporter construct expression in control testes, and no signal in *Nxt1^Z2-0488^*/*Nxt1^DG05102^* or *comr* mutant testes. Addition of one intron generates detectable, although weak, expression in *Nxt1^Z2-0488^*/*Nxt1^DG05102^* testes, but not in *comr* mutant testes.

To test whether this finding is a general property of tMAC-dependent promoters we generated a reporter using the *CG42355* promoter and 5′ UTR. *CG42355* is expressed specifically in testes in the same pattern as *djl*. *CG42355* expression depends on tMAC but less strongly on *Nxt1* (Figure 3 F–F′″, Figure 8C). *CG42355* promoter + 5′UTR (−177/+136)- LacZ reporter lines recapitulated the native expression of the mRNA in control testes (Figure 8B), and revealed that *CG42355*, like *djl*, is significantly translationally repressed (Figure S3). Expression of this construct was barely detected in both *comr* and *Nxt1* mutant spermatocytes (Figure 8C, Figure 9). We conclude that the tMAC-dependence of a promoter + 5′UTR is sufficient to confer *Nxt1*-dependence onto an exogenous transcript. The native *CG42355* gene must contain features that allow the transcript to overcome this requirement for Nxt1, and be expressed at only 5× reduced levels in *Nxt1* mutant cells.

### Translational repression does not confer Nxt1 dependence

tMAC predominantly regulates genes whose transcripts are normally translationally repressed in the male germline; if Nxt1 is required to recognise and protect translationally repressed mRNAs then the overlap in target genes would be merely co-incidental. To test this we used a *djl+43-LacZ* reporter construct (Figure 8A), which is transcribed in primary spermatocytes, but not translationally repressed (Figure S3) [32]. We confirmed by both RNA *in situ* hybridisation (data not shown) and q-RT-PCR (Figure 8C) that transcription of this reporter depends on tMAC function. Expression of this reporter in *Nxt1* mutant testes was even lower than expression of the *djl+95-LacZ* construct, indicating that the promoter and first 43 bp of 5′UTR, and not the translational repression sequence of the mRNA, is responsible for conferring *Nxt1* dependence on this exogenous reporter transcript (Figure 8B, C). This correlates with the results obtained from the UAS-djl+95-LacZ construct, in which an mRNA that is translationally repressed accumulates normally in *Nxt1* mutant testes (Figure 8B, Figure S3).

### Short and intron-less genes are particularly sensitive to the lack of *Nxt1* function

To understand why some tMAC-dependent transcripts require *Nxt1* for their expression, while others show much lower dependence on *Nxt1*, we further analysed the microarrays, and focused on genes 16× or more down-regulated in *Nxt1* or *aly* mutants. Highly *aly*-dependent transcripts were significantly longer (mean 1694 bp, SD 1209) than highly *Nxt1*-dependent transcripts (mean 1518 bp, SD 1170) (2-tailed Mann-Whitney U test, p = 0.0024). This difference was even more dramatic when the length of highly *Nxt1*-dependent transcripts was compared to those that depend highly on *aly* but are not 16× or more down-regulated in *Nxt1* (mean 1800 bp, SD 1238) (2-tailed Mann-Whitney U test, p<0.0001). Thus we conclude that shorter transcripts are more likely to be dramatically under-expressed in *Nxt1* testes than longer transcripts. However *CG42355* encodes a short transcript (<1 kb) that is only mildly down-regulated in *Nxt1* mutant testes, while the LacZ reporter driven by this promoter is much longer (>3 kb), and is dramatically under-expressed in the mutant, so other features of the transcript must also be implicated.

Approximately 22% of all annotated *Drosophila* genes lack introns. 1412 genes are expressed specifically in testis (based on FlyAtlas data) and 521 (37%) of these lack introns, a significantly higher proportion than expected by chance (Chi-squared, p<0.0001). Thus, intron-less genes are enriched for testis-specific expression. Of the 1019 genes that were 16× or more reduced in *aly* testes, 404 (40%) lack introns, indicating that the presence or absence of introns does not affect whether a testis-specific transcript is also highly *aly*-dependent (Chi-squared, p = 0.18). In contrast, of the 456 highly *Nxt1*-dependent genes, 303 (66%) lack introns, revealing that highly *Nxt1*-dependent genes are significantly more likely to lack introns than randomly selected testis-specific genes (Chi-squared, p<0.0001). Genes that are highly dependent on both *aly* and *Nxt1* for their expression are predominantly intron-less while genes that are highly dependent on *aly*, but are only mildly dependent on *Nxt1* predominantly have introns (Figure 6D). Notably, the more introns present in a strongly tMAC-dependent gene, the higher its expression in an *Nxt1* background (Figure S4). We analysed the 300 most down-regulated genes in a tMAC mutant, and correlated fold change compared to control in *Nxt1* mutants with intron number (0, 1, 2, 3+). Relative expression of genes lacking introns was significantly lower than that of genes containing introns (t-test, p = 1.18889E-32). Relative expression of genes with 1 intron was not significantly different than that of 2-intron genes (t-test, p = 0.126), however genes with 1 or 2 introns had significantly lower expression in *Nxt1* mutants compared to wild type than genes with 3 or more introns (t-test, p = 0.00013). This indicates a dose-response relationship between gene expression in *Nxt1* mutant testes and intron number, where the more introns a tMAC-dependent gene, has the lower its requirement for *Nxt1* for expression.

### The presence of introns partially negates the requirement for Nxt1 in reporter gene expression

The microarray results indicate that having at least one intron is beneficial for expression of tMAC-dependent genes in *Nxt1* mutants. We used reporter constructs to test how the presence or absence of introns impacts on reporter gene expression in *Nxt1* testes (Figure 8A). We inserted the *djl* intron into the 5′UTR of (the previously intron-less) *djl* −555/+95 reporter sequence, and found that the reporter was testis-specifically expressed, and translation was delayed until spermatid elongation stages, in a pattern indistinguishable from the original construct. Similarly, insertion of the CG42355 introns into the LacZ coding sequence of the *djl* construct resulted in expression mirroring the original reporter (Figure S3). Equivalent one and two intron CG42355 reporters were constructed (Figure 8A). We confirmed correct splicing of these transcripts by RT-PCR (data not shown).

When we tested expression of these intron-containing reporters in *Nxt1* spermatocytes we found that insertion of the introns partially restored the reporter expression (Figure 8D, Figure 9). Strikingly, the *djl*- reporter with two introns was expressed at over 50% of wild type levels. Similarly, introduction of two introns into the *CG42355* reporter elevated expression to 15% of that seen for the reporter in wild type spermatocytes. This represents a 10× increase in expression in mutants compared to the intron-less version of the reporter. Insertion of introns did not increase reporter expression in *comr* mutant testes (Figure 9). We conclude that, in spermatocytes, tMAC-dependent promoters confer dependence on *Nxt1* for transcript expression, but that this dependence can be partially overcome if the transcript has one or more introns.

## Discussion

### Identification of Nxt1 as a novel regulator of gene expression in the male germline

To date 14 meiotic arrest loci have been described (*aly*, *achi-vis*, *topi*, *tomb*, *comr*, *wuc*, *mip40*, *can*, *mia*, *sa*, *rye*, *nht*, *thoc5*, *Nurf301*) [15], [28], [33]. Most of these encode subunits of either a testis-specific TFIID complex or tMAC. The exceptions are mutations resulting in a C-terminally truncated product from *Nurf301* [33], and loss of *thoc5* [28]. In both these cases the effect on gene expression is apparently much less dramatic than loss of either tTAFs or tMAC. Here we identify *Nxt1* as a novel meiotic arrest locus, and show that the phenotype does not fit with any previous classification. Notably, mutation of *Nxt1* has much more dramatic effects on gene expression than *thoc5*, *Nurf301* or tTAFs, although the defect is not as pronounced as that seen in most tMAC mutants.

Nxt1 protein acts in the same biochemical pathway as the TREX subunit, Thoc5, suggesting that the entire RNA export pathway might be critical for testis gene expression. In support of this, in preliminary experiments we have observed incomplete meiotic arrest phenotypes in testes with spermatocyte specific knock down (by RNAi) of either *Ref1* or *sbr* (data not shown). No defects in gene expression were detected in the *thoc5* mutant [28], but we note that the genes assayed (*bol*, *twe*, *cycB*, *dj*, *fzo*, *Mst87F*) all contain at least one intron, and, although *fzo*, *bol* and *dj* have reduced expression in *Nxt1* testes, none is on the list of genes 16× or more down regulated in this background. It would be very interesting to determine whether the intron-less genes most dependent on *Nxt1* for expression are also dependent on *thoc5* or other RNA export factors. Notably, Nxt1 and Thoc5 localise to a punctate structure adjacent to the nucleolus in early primary spermatocytes and to a structure adjacent to the nuclear envelope in late primary spematocytes. This localisation adjacent to the nuclear envelope is also seen for Nxf1, the binding partner of Nxt1, encoded in *Drosophila* by the *sbr* gene [28], [30], [34]. Both Nxt1 and Sbr also localise to one face of the nucleus in spermatids. This co-localisation suggests that the proteins are all working together in these cells.

### A model to explain how tMAC and the RNA export factors together regulate testis gene expression

The direct link we describe here, between tMAC and RNA export in regulating testis gene expression, can be explained by two theoretical mechanisms. (1) tMAC could promote transcription of target genes and feed-forward to promote their stability and RNA export. In the absence of Nxt1 the mRNAs would not be protected, and instead would be degraded. (2) Initially low tMAC activity promotes production of low levels of transcript. Binding of the export factors to these transcripts during processing would feed-back to increase the activity of tMAC. This would then increase the transcription of the tMAC-dependent gene. The ability of introns to rescue expression in the mutant indicates that the mechanism must be mediated through the RNA transcript, and presumably depends on the higher affinity of spliced transcripts for the Nxt1/Nxf1 dimer. If the feed-forward mechanism predominates, the rate of nascent transcript production should be equal to wild type. If feed-back predominates, the rate of nascent transcript production would be lower than wild type. We found a reduction in both nascent and mature transcripts in the mutant, although the reduction in mRNA is greater than the reduction in nascent RNA. We propose that both feed-forward and feedback occur, to give an amplification loop. This amplification would depend on a specific interaction between the RNA export factors and tMAC.

### The RNA nuclear export pathway and tissue specific transcript accumulation

The RNA nuclear export pathway has been deduced primarily using experiments in yeast, and in tissue culture cells (reviewed in [1]). *Drosophila* culture cells depleted of components of the pathway, including *Nxt1*, accumulate poly-adenylated mRNAs within the nucleus. Most transcripts in the cell are also reduced in abundance, typically a 1.5–2 fold level change for altered transcripts [35]. Very few transcripts were reduced more than 5 fold after RNAi treatment, and very few were significantly increased in abundance. The RNAi treatments used in these experiments also caused defects in cell proliferation or viability. The *in vivo* role of *sbr*, which encodes the Nxf1 partner of Nxt1, has been investigated in embryos and larvae, and again defects have been detected in bulk export of mRNAs from nuclei [36]. In none of these experiments has a link been detected between the role of the Nxt1/Nxf1 and transcription.

Factors in the mRNA export pathway, upstream of Nxt1/Nxf1 have been linked to transcription, consistent with the fact that RNA splicing and processing is co-transcriptional. Specifically the TREX complex interacts with chromatin and RNA polymerase II, and is important for facilitating transcription elongation, 3′ end formation, mRNA processing, and transfer to the nuclear pore [4]. While the interaction of TREX with RNA polymerase is well established, there has been no data to implicate Nxt1/Nxf1 in this process. TREX promotes association of REF with the transcript, and this in turn promotes Nxt1/Nxf1 association [37]. Similarly, the spliceosome can have a stimulatory effect on transcription, both at the level of initiation and elongation [38]. Our study is the first, to our knowledge, to implicate these pathways in the regulation of a programme of gene expression promoted by a specific transcription factor complex.

Recently two studies have demonstrated that the stability of specific *S. cerevisiae* mRNAs can depend on non-transcribed promoter sequences. RPL30 mRNA was shown to have a short half-life imposed by the transcriptional activator Rap1 and its binding site in the UAS, although how this affects the RNA is not yet determined [39]. The cell cycle regulation of the half-lives of SWI5 and CLB2 was independent of the transcript sequences, was coordinated with transcription, and was promoter dependent [40]. Dbf2, a kinase, is recruited by specific promoter sequences and co-transcriptionally deposited on the SWI5 and CLB2 mRNAs. Dbf2 is a component of the CCR4-NOT cytoplasmic deadenylase complex, and the activity of this complex could then normally de-stabilise these transcripts. In both these examples the promoter acts to confer a short half-life on the transcripts it regulates. We report a similar phenomenon in an animal system, however the outcome is increased mRNA expression, with the RNA processing pathway feeding back to increase transcription.

### Why don't transcripts accumulate in *Nxt1* mutant nuclei?

The canonical role for Nxt1 is export of mRNAs from the nucleus, however, paradoxically, and in contrast to the results from tissue culture studies [41], we do not detect accumulation of mRNAs in *Nxt1* mutant nuclei. The majority of mRNAs expressed in the mutant spermatocytes are present at normal levels, and are detected in the cytoplasm. This mRNA export capability could be provided by residual Nxt1 function, or by a parallel pathway. We suggest instead that residual Nxt1 function provided by the hypomorphic allele is sufficient for mRNA export. This interpretation is supported by the observation that the null allele is embryonic lethal while the z2-0488 allele is viable, thus sufficient activity remains in this allele to support normal function of most cells. The fact that reduction in Nxt1 levels by RNAi in spermatocytes phenocopies the z2-0488 allele indicates that the effect is due to reduced activity, rather than an allele-specific effect. A well characterised alternative mRNA export pathway involves the Crm1 protein, binding RNA via an unidentified adaptor protein. Crm1 is implicated in export of a subset of endogenous RNAs, as well as HIV mRNA in humans [42]. The *Drosophila* Crm1 protein is encoded by the gene *embargoed*. This gene is expressed at much lower levels in testes than in other adult tissues, and thus is unlikely to represent the major RNA export pathway in spermatocytes, given their high level of transcriptional activity.

### How does the presence of introns promote transcript accumulation in *Nxt1* mutant spermatocytes

Nxt1/Nxf1 heterodimers are already known to be implicated in export of some mRNAs with retained introns, and have been demonstrated to interact with constitutive transport elements present in some cellular and viral mRNA [43]. Similarly, in human cells naturally intronless transcripts tested were transported from the nucleus in a TREX and Nxf1 dependent manner [44]. In the absence of Nxf1 the transcripts remained nuclear, but apparently were not destabilised. When nascent transcripts are being produced they are bound by hnRNPs, and those with introns are processed by the spliceosome. During this processing the EJC associates with the transcript. This is then responsible for recruitment of Ref1, which recruits Nxt1/Nxf1. Transcripts that lack introns also associate with Ref1 and thus Nxt1/Nxf1, but without the help of the EJC. Thus the EJC increases the affinity of Nxt1/Nxf1 to the transcript. For a transcript being synthesised in an Nxt1-depleted background this could be sufficient to ensure association of the export factor, and thus to ensure proper processing and export. We detect a dose response for intron number, specifically, the more introns a tMAC dependent gene has the higher its expression in an *Nxt1* mutant background. If, as we propose, there is feedback from the export pathway to increase tMAC activity, then the presence of the EJC and the increased affinity for Nxt1/Nxf1 would result in higher transcript levels from intron-containing genes than intron-less genes in an Nxt1-depleted background, consistent with our findings.

### Introns and evolution of testis-enriched genes

It is well demonstrated that the presence of introns in primary transcripts correlates with higher gene expression levels [45]; this is true for almost all *Drosophila* adult tissues. Indeed, most ectopic expression systems include at least one intron to facilitate higher transcription levels, higher transcript stability and higher translation efficiency. However the testis is unique among *Drosophila* adult tissues in that, amongst genes whose expression is detected, the expression level of intron-containing genes is significantly lower than that of intron-less genes (Mann-Whitney U test, p<0.001) (deduced from FlyAtlas data; expression is in arbitrary units). Specifically, the median expression signal for intron-containing genes in brain (a typical somatic tissue) is 139, while that for intron-less genes is 78.1. In contrast, the equivalent figures for testes are 84 and 168.5. In testis, and in somatic tissues, approximately 60–65% of all annotated intron-containing protein coding genes are expressed. In somatic cells only 30–35% of all annotated intron-less genes are expressed. In contrast, in testes, 63% of all annotated intron-containing genes are expressed (Figure S5). Thus spermatocytes must have evolved a mechanism to support high level expression of intron-less genes. Many of the intron-less genes expressed in testes are retroposed copies of intron-containing genes. Typically the parent gene will have a broad or ubiquitous expression pattern while the retroposed gene's expression is highly restricted [46]. The new gene is often subject to rapid evolution under positive selection, and contributes to sperm-specific cell biology. Most of these retroposed gene copies are regulated by the tMAC transcriptional regulation complex, and, like other tMAC-dependent genes, they typically have short promoters. Surprisingly, the promoter regions, and even transcription start sites, are not highly conserved between species [47], although the insertions are biased towards genomic regions already containing testis-specific transcription units [48]. This could be explained by low-affinity tMAC binding occurring by virtue of the chromosomal domain, promoting transcription of the newly inserted sequence, and feedback from RNA export factors serving to increase tMAC activity, and thus expression of the new gene.

### Nxt1 function is not limited to the male germline

In this study we have demonstrated a link between the core RNA export pathway and testis-specific transcription. Null alleles of *Nxt1* are recessive lethal, and the hypomorph (homozygous or hemizygous has low viability. The animals often pupate with a distinctive elongated curved shape, uneverted spiracles, and then fail in head eversion, consistent with defects in air bubble movement. These processes are controlled by transcriptional responses downstream of ecdysone signalling, and the mutant phenotype is highly reminiscent of mutants in *Eip74EF*, an ets family transcription factor [49]. Nxt1^z2-0488^ homozygous or hemizygous adult females are also sterile. These highly reproducible defects potentially also arise as a result of defects in gene expression in the relevant tissues, rather than being caused by a general defect in export of all mRNAs from the nuclei of somatic or female germline cells. In testes we have demonstrated that the effect is via a specific transcription complex, tMAC. The soma and female germline defects cannot be caused by an interaction between Nxt1 and tMAC since tMAC expression is restricted to testes. We postulate that Nxt1 might be important for regulating the transcriptional response to ecdysone during pupariation, and expression of specific genes in the female germline. Thus we suggest that Nxt1 could work with, as yet unidentified, specific transcription factors to control gene expression in other tissues.

## Materials and Methods

### Cloning of *z2-0488*

We mapped the male and female sterility, and semi-lethality, of *z2-0488* using deficiency chromosomes to a region of approximately 100 kb containing 22 known or predicted genes at the distal end of chromosome 2R (Figure S1). 9 candidate genes were sequenced, and a single non-synonymous change was found (D126N, codon GAT-AAT) in the *Nxt1* gene. A P-element insertion allele of *Nxt1*, *P{wHy}DG05102*, failed to complement the semi-lethality and sterility of the *z2-0488* mutation. This P-element insertion, which is in the first exon, just downstream of the ATG, and predicted to be a null allele, results in homozygous and hemizygous lethality. We conclude that *z2-0488* is a hypomorphic allele of *Nxt1*, and designate the allele *Nxt1^z2-0488^*.

### Microscopy and in situ hybridisation

Testes were dissected from young adults, prepared for phase contrast and fluorescence microscopy as in [50] and imaged using a Hamamatsu Orca-05G camera driven by HCImage software on an Olympus BX50 microscope. RNA in situ hybridisation using dig-labelled RNA probes was as in [51]. Primer sequences used to generate templates are in Text S1. *CycB* probe was made from a cDNA clone as in [17]. Templates for *CG42355* and *djl* were subcloned into pGEM-T-Easy, while other probes were generated directly from PCR products. Beta-galactosidase activity assays on whole mount testes were as in [17]. Colour images were taken with a JVC-F75U camera run by KY-LINK software mounted on the Olympus BX50 microscope. Image composites were assembled using Adobe Photoshop.

### Plasmid construction

djl-lacZ −555/+95 and djl-lacZ −555/+43 [32] were provided by Renate Renkawitz-Pohl. PCR from genomic DNA yielded a fragment 5′-EcoRI-CG42355-177/+136-BamHI-3′ which was cloned into pCaSpeR4-AUG-betaGAL [52] to yield CG42355-LacZ −177/+136. The plasmids djl-lacZ -555/+95 +1 intron, djl-lacZ −555/+95 +2 introns, djl-lacZ −555/+43 +1 intron, djl-lacZ −555/+43 +2 introns, CG42355-lacZ −177/+136 +1 intron and CG42355-lacZ +2 introns were derived from these basic promoter-reporter constructs by incorporation of a synthetic DNA fragment containing the intron(s) (see Text S1 for sequences and construction details). pUAST-eGFP-Nxt1 and pUAST-eGFP-Nxt1-D126N were generated by PCR and subcloning of the Nxt1 ORF from *Nxt1^z2-0488^*/CyO flies [53]. pCaSpeR4-UAS-djl- AUG-betaGAL was made by cloning a synthetic fragment comprising 5× UAS; Hsp70 minimal promoter (up to the TSS); +1–+95 of djl into the MCS of pCaSpeR4-AUG-betaGAL using EcoRI and BamH1 (Text S1).

### *Drosophila* methods

*Drosophila* were maintained on cornmeal, sucrose, yeast, agar medium, at 25°C (29°C for RNAi crosses). Mutant alleles were *aly^5^*, *comr^Z1340^*, *nht^Z2-5946^*, *Nxt1^DG05102^*, *Nxt1^z2-0488^*. *w^1118^* was used as a control. For RNAi against *Nxt1* we used UAS-hairpin lines from the Vienna Drosophila Resource Centre [54] driven by bam-Gal4VP16, with both constructs heterozygous. bam-Gal4VP16 was also used to drive fusion protein constructs in spermatocytes. tub-Gal4 was from the Bloomington Drosophila stock centre and was used to drive ubiquitous fusion protein expression. Transgenic lines were selected after injection of P-element constructs into *w^1118^* using standard methods. Insertions on the third chromosome were selected, balanced, and crossed into *Nxt1^z2-0488^*, *Nxt1^DG05102^* and *comr^Z1340^* mutant backgrounds.

### Purification of RNA from spermatocytes

For q-RT-PCR, total RNA was extracted from purified spermatocyte samples using the RNAqueous micro kit (Invitrogen). Testes were dissected from 1–3 adult males of the appropriate genotype in TB (183 mM KCl, 47 mM NaCl, 10 mM Tris pH7.4), transferred to a small drop of TB on a hydrophobic plate, and the testis tip was cut open using a tungsten needle. Spermatocytes were pushed out of the testis sheath into the buffer with the needle and the remainder of the testis discarded. Most of the buffer was removed, leaving approximately 1–2 µl, and 10 µl lysis solution was added. The lysate was added to 90 µl lysis solution in a microfuge tube; immediately frozen in liquid nitrogen, and stored at −80°C. Preparation of each sample took no more than 10 minutes. RNA was purified according the manufacturer's instructions. RNA was eluted from the spin column with 2×6.5 µl of elution buffer. The total eluate volume was approximately 11 µl.

### RT-PCR and q-RT-PCR

For conventional q-RT-PCR, cDNA was synthesised using Superscript III (Invitrogen) and oligo-dT primers. For analysis of nascent RNA and spliced products the purified RNA was treated with DNase I, half of the RNA sample was reserved as a control to assay DNA contamination and the remainder was reverse transcribed using Superscript III and random hexamer primers. PCR primers (sequences in Text S1) were used that recognised exon, intron or exon-exon junction sequences. The cDNA reaction was diluted to 60 µl with water, and 1 µl of this was used as a template in PCR reactions, using PowerSybr reagent (ABI) in a Chromo4 instrument (MJR). For q-RT-PCR of reporter gene expression and *CG42355* and *djl* levels, the entire RNA eluate was used for cDNA synthesis with oligo dT primers. *CG3927* was used as a control gene for relative quantitation using the ΔΔCt method. Expression of the test gene relative to *CG3927* gene was normalised to 1 in the control sample. Control testes had the relevant transgene present in the same copy number as the test sample, but had normal testis morphology. *CG3927* expression is restricted to primary spermatocytes (Figure 3). *CG3927* expression in *Nxt1* and *comr* mutant testes is similar to wild type, as judged by both RNA in situ hybridisation and microarray analysis. All reactions were performed in triplicate. We performed biological replicates for the djl+95 and djl+43 lines, for which only one insertion was available. For other reporters we used two different third-chromosome insertion lines, and show both results.

### Microarray and bioinformatics

Testes from *Nxt1^z2-0488^*/*Nxt1^DG05102^* transheterozygote males, raised at 25°C were used for Affymetrix microarray analysis, and were compared to our existing microarray data sets. Sample preparation, processing and data analysis were as described in [16]. The FlyMine interface was used to extract lists of probes associated with intron-containing and intron-less genes, as well as to extract data from FlyAtlas on gene expression profiles in adult and larval tissues. A list of 1412 genes with testis-specific expression (1523 probes) was created by filtering of FlyAtlas data [11]. We selected those probes where the present call in testis was 4/4, and sum of present calls in all other adult tissues was <4.

FlyMine (v27.0, Feb 2011) was used to generate lists of genes with and without introns. First a list of all annotated *Drosophila melanogaster* genes was created. A list of genes with introns were selected using the “Gene → Introns” template, and selecting the column “genes” as output. A list of all genes without introns was generated by subtracting the genes with introns list from the all genes list. The “Gene → Affymetrix probe” template was used to generate a list of all probes for intron-less genes. This was then combined with our Affymetrix data in Microsoft Excel to allow us to analyse intron-containing and intron-less genes separately. The number of introns present in each of the 300 genes most down regulated in *tomb* was determined by first ranking Affymetrix fold change data for *tomb*-vs.-control, then manually checking annotations in FlyBase. Annotated transcripts were cross-referenced with the RNA-seq data for adult males to ensure accuracy. All genes with valid male- (probably testis-) expressed spliced transcripts were scored as having an intron, even if they also encoded alternative intron-less transcripts.

## Supporting Information

Figure S1 Cloning of Z2-0488. The meiotic arrest phenotype of Z2-0488 was mapped by recombination to a region of chromosome 2R. From published data [55] we defined the proximal end of the Z2-0488 region by the distal breakpoint of Df(2R)bw5, which uncovers *egl*, but does not uncover Z2-0488; and by the proximal breakpoint of Df(2R)or-BR11, which uncovers Z2-0488 but not *egl*. The distal end of the Z2-0488 region was defined by the proximal breakpoint of Df(2R)HB132, which uncovers *egl* and Z2-0488, but does not uncover *gbb*. Thus Z2-0488 lies between *egl* and *gbb*. The lethality of *P{wHy}Nxt1^DG05102^* had the same complementation pattern with respect to deficiencies as Z2-0488. Candidate genes from the region were sequenced (blue), and the only mutation detected in Z2-0488 was in *Nxt1*.
(PDF)

Figure S2 *Nxt1*-dependent transcripts are also highly *aly*-dependent but not vice-versa. Scatter plots of microarray data comparing gene expression in wild type with *Nxt1* mutant (A, C, E) or with *aly* mutant (B, D, F). Probes that pass the threshold filters (16×, 8× or 4× down in mutants vs. wild type) are coloured (A, B). (C, D) expression plots as in A or B are recoloured according to how the probes behave in the other mutant genotype. Probes in purple in panel D (wt vs. *aly*) are those whose expression is 16 fold or more reduced in *Nxt1* mutants compared to wild type. These all cluster in the bottom right hand corner of the plot, ie they have high expression in wild type and low expression in *aly*. Probes in red in panel C (wt vs. *Nxt1*) are those whose expression is 16 fold or more reduced in *aly* mutants compared to wild type. These spread up the right hand side of the plot, ie they have high expression in wild type, and high, medium or low expression in *Nxt1* mutants. (E, F) The same plots as C and D except that the probes that did not pass the filters (ie less than 4× change), coloured white, are plotted on top of the other data rather than behind. Very few white dots are present in the bottom right corner in panel E while many white dots are in this region in F. This demonstrates that virtually all genes that depend on *Nxt1* also depend on *aly*, but that not all genes that depend on *aly* also depend on *Nxt1*. The expression of known meiotic arrest genes in mutant testes, is shown by large dots in panels A and B. None of the known meiotic arrest genes have reduced expression in *Nxt1* or *aly* testes.
(PDF)

Figure S3 Reporter construct LacZ protein expression patterns. Beta-galactosidase activity staining reveals that translation of both djl+95-LacZ and 42355-LacZ is delayed until spermatid differentiation, while djl+43-LacZ translation is detected in late primary spermatocytes. Insertions of introns into the reporters does not alter the translation timing or protein expression pattern. Expression of the djl+95-LacZ transcript with the UAS construct results in earlier expression than of the endogenous transcript. There is a bi-phasic protein expression profile, with translation in early primary spermatocytes, repression in later primary spermatocytes and early spermatids, and a second wave of translation in spermatids. When expressed in *Nxt1^Z2-0488^*/*Nxt1^DG05102^* testes the protein is expressed in early primary spermatocytes, and repressed in the later primary spermatocytes.
(TIFF)

Figure S4 Effect of intron number on TMAC-dependent gene expression in *Nxt1^Z2-0488^*/*Nxt1^DG05102^* testes. The scatter plot shows the 300 transcripts most down-regulated in *tomb* mutant testes. The array data of log2 transformed relative expression of transcripts in TMAC mutant testes (*tomb*) compared to wild type is plotted on the y-axis, while the relative expression in *Nxt1^Z2-0488^*/*Nxt1^DG05102^* testes compared to control is on the x-axis. Transcripts from genes with no introns are indicated by blue squares, while those from genes with 1, 2 or 3 or more are indicated with green, orange and red diamonds respectively. Mean expression fold changes are show with large dots.
(TIF)

Figure S5 Expression of genes with and without introns in adult and larval fly tissues. The proportion of all annotated genes with (blue) or without introns (red) whose expression is detected in a specific tissue. Only testis expresses a the same proportion of intron-less genes as intron containing genes. br = brain; hg = hindgut; mg = midgut; sg = salivary gland; tt = testis; he = heart; wf = whole fly; cr = crop; hd = head; tg = thoracio-abdominal ganglion; ac = adult carcass; ae = eye; af = adult fatbody; ma = male accessory gland; st = S2 cells; tu = malpigian tubule; vs. = virgin female spermatheca; ms = mated female spermatheca; lh = larval hindgut; lc = larval CNS; lk = larval carcass; lm = larval midgut; ls = larval salivary gland; lt = larval trachea; lu = larval malpigian tubule.
(TIF)

Text S1 This file contains details of the cloning of introns into the reporter constructs, including the sequences used in their native context and in the reporter construct context. This file also contains sequences for all the PCR primers used in the manuscript.
(DOC)

## References

[1] Carmody SR, Wente SR (2009) mRNA nuclear export at a glance. J Cell Sci 122: 1933–1937. 19494120 10.1242/jcs.041236 PMC2723150

[2] Erkmann JA, Kutay U (2004) Nuclear export of mRNA: from the site of transcription to the cytoplasm. Exp Cell Res 296: 12–20. 15120988 10.1016/j.yexcr.2004.03.015

[3] Jimeno W, Rondón A, Luna R, Afuilera A (2002) The yeast THO complex and mRNA export factors link RNA metabolism with transcription and genome instability. EMBO J 21: 3526–3535. 12093753 10.1093/emboj/cdf335 PMC126085

[4] Strässer K, Masuda S, Mason P, Pfannstiel J, Oppizzi M, et al (2002) TREX is a conserved complex coupling transcription with messenger RNA export. Nature 417: 304–308. 11979277 10.1038/nature746

[5] Le Hir H, Gatfield D, Izaurralde E, Moore M (2001) The exon-exon junction complex provides a binding platform for factors involved in mRNA export and nonsense-mediated mRNA decay. EMBO J 20: 4987–4997. 11532962 10.1093/emboj/20.17.4987 PMC125616

[6] Zhou Z, Luo M, Straesser K, Katahira J, Hurt E, et al (2000) The protein Aly links pre-messenger-RNA splicing to nuclear export in metazoans. Nature 407: 401–405. 11014198 10.1038/35030160

[7] Strässer K, Hurt E (2000) Yra1p, a conserved nuclear RNA-binding protein, interacts directly with Mex67p and is required for mRNA export. EMBO J 19: 410–420. 10722314 10.1093/emboj/19.3.410 PMC305578

[8] Abruzzi K, Lacadie S, Rosbash M (2004) Biochemical analysis of TREX complex recruitment to intronless and intron-containing yeast genes. EMBO J 23: 2620–2631. 15192704 10.1038/sj.emboj.7600261 PMC449771

[9] Watanabe M, Fukuda M, Yoshida M, Yanagida M, Nishida E (1999) Involvement of CRM1, a nuclear export receptor, in mRNA export in mammalian cells and fission yeast. Genes Cells 4: 291–297. 10421839 10.1046/j.1365-2443.1999.00259.x

[10] Andrews J, Bouffard GG, Cheadle C, Lu JN, Becker KG, et al (2000) Gene discovery using computational and microarray analysis of transcription in the *Drosophila melanogaster* testis. Genome Research 10: 2030–2043. 11116097 10.1101/gr.10.12.2030 PMC313064

[11] Chintapalli V, Wang J, Dow J (2007) Using FlyAtlas to identify better *Drosophila* models of human disease. Nat Genet 39: 715–720. 17534367 10.1038/ng2049

[12] Olivieri G, Olivieri A (1965) Autoradiographic study of nucleic acid synthesis during spermatogenesis in *Drosophila melanogaster* . Mutat Res 2: 366–380. 5878312 10.1016/0027-5107(65)90072-2

[13] Zhao J, Klyne G, Benson E, Gudmannsdottir E, White-Cooper H, et al (2010) FlyTED: the *Drosophila* testis gene expression database. Nucleic Acids Research 38: D710–715. 19934263 10.1093/nar/gkp1006 PMC2808924

[14] Schäfer M, Nayernia K, Engel W, Schäfer U (1995) Translational control in spermatogenesis. Developmental Biology 172: 344–352. 8612956 10.1006/dbio.1995.8049

[15] White-Cooper H (2010) Molecular mechanisms of gene regulation during *Drosophila* spermatogenesis. Reproduction 139: 11–21. 19755484 10.1530/REP-09-0083

[16] Doggett K, Jiang J, Aleti G, White-Cooper H (2011) Wake-up-call, a *lin-52* paralogue, and Always early, a *lin-9* homologue physically interact, but have opposing functions in regulating testis-specific gene expression. Dev Biol 355: 381–393. 21570388 10.1016/j.ydbio.2011.04.030 PMC3123737

[17] White-Cooper H, Schafer MA, Alphey LS, Fuller MT (1998) Transcriptional and post-transcriptional control mechanisms coordinate the onset of spermatid differentiation with meiosis I in *Drosophila* . Development 125: 125–134. 9389670 10.1242/dev.125.1.125

[18] Beall EL, Lewis PW, Bell M, Rocha M, Jones DL, et al (2007) Discovery of tMAC: a *Drosophila* testis-specific meiotic arrest complex paralogous to Myb-MuvB. Genes & Development 21: 904–919. 17403774 10.1101/gad.1516607 PMC1847709

[19] Wang Z, Mann RS (2003) Requirement for two nearly identical TGIF-related homeobox genes in *Drosophila* spermatogensis. Development 130: 2853–2865. 12756170 10.1242/dev.00510

[20] Ayyar S, Jiang J, Collu A, White-Cooper H, White R (2003) *Drosophila* TGIF is essential for developmentally regulated transcription in spermatogenesis. Development 130: 2841–2852. 12756169 10.1242/dev.00513

[21] Jiang J, Benson E, Bausek N, Doggett K, White-Cooper H (2007) Tombola, a tesmin/TSO1 family protein, regulates transcriptional activation in the *Drosophila* male germline and physically interacts with Always early. Development 134: 1549–1559. 17360778 10.1242/dev.000521 PMC2229809

[22] Jiang J, White-Cooper H (2003) Transcriptional activation in *Drosophila* spermatogenesis involves the mutually dependent function of aly and a novel meiotic arrest gene *cookie monster* . Development 130: 563–573. 12490562 10.1242/dev.00246

[23] Perezgazga L, Jiang J, Bolival B, Hiller MA, Benson E, et al (2004) Regulation of transcription of meiotic cell cycle and terminal differentiation genes by the testis-specific Zn finger protein *matotopetli* . Development 131: 1691–1702. 15084455 10.1242/dev.01032

[24] White-Cooper H, Leroy D, MacQueen A, Fuller MT (2000) Transcription of meiotic cell cycle and terminal differentiation genes depends on a conserved chromatin associated protein, whose nuclear localisation is regulated. Development 127: 5463–5473. 11076766 10.1242/dev.127.24.5463

[25] Chen X, Hiller MA, Sancak Y, Fuller MT (2005) Tissue-specific TAFs counteract Polycomb to turn on terminal differentiation. Science 310: 869–872. 16272126 10.1126/science.1118101

[26] Hiller MA, Chen X, Pringle MJ, Suchorolski M, Sancak Y, et al (2004) Testis-specific TAF homologs collaborate to control a tissue-specific transcription program. Development 131: 5297–5308. 15456720 10.1242/dev.01314

[27] Hiller MA, Lin T-Y, Wood C, Fuller MT (2001) Developmental regulation of transcription by a tissue-specific TAF homolog. Genes and Development 15: 1021–1030. 11316795 10.1101/gad.869101 PMC312677

[28] Moon S, Cho B, Min S-H, Lee D, Chung YD (2011) The THO complex is required for nucleolar integrity in *Drosophila* spermatocytes. Development 138: 3835–3845. 21828100 10.1242/dev.056945

[29] Koundakjian E, Cowan D, Hardy R, Becker A (2004) The Zuker collection: a resource for the analysis of autosomal gene function in *Drosophila melanogaster* . Genetics 167: 203–206. 15166147 10.1534/genetics.167.1.203 PMC1470872

[30] Herold A, Klymenko T, Izaurralde E (2001) NXF1/p15 heterodimers are essential for mRNA nuclear export in *Drosophila* . RNA 7: 1768–1780. 11780633 PMC1370216

[31] Fribourg S, Braun IC, Izaurralde E, Conti E (2001) Structural basis for the recognition of a nucleoporin FG repeat by the NTF2-like domain of the TAP/p15 mRNA nuclear export factor. Molecular Cell 8: 645–656. 11583626 10.1016/s1097-2765(01)00348-3

[32] Hempel L, Rathke C, Raja S, Renkawitz-Pohl R (2006) In *Drosophila*, *don juan* and *don juan like* encode proteins of the spermatid nucleus and the flagellum and both are regulated at the transriptional level by the TAF_II_80 cannonball while translational repressio is achieved by distinct elements. Developmental Dynamics 235: 1053–1064. 16477641 10.1002/dvdy.20698

[33] Kwon S, Xiao H, Wu C, Badenhorst P (2009) Alternative splicing of NURF301 generates distinct NURF chromatin remodelling complexes with altered modified histone binding specificities. PLoS Genet 5: e1000574. 19629165 10.1371/journal.pgen.1000574 PMC2705796

[34] Atsapkina A, Golubkova E, Kasatkina V, Avanesyan E, Ivankova N, et al (2010) Peculiarities of spermatogenesis in *Drosophila melanogaster*: role of main transport receptor of mRNA (Dm NXF1). Cell and Tissue Research 4: 1–7.

[35] Herold A, Teixeira L, Izaurralde E (2003) Genome-wide analysis of nuclear mRNA export pathways in *Drosophila* . EMBO J 22: 2472–2483. 12743041 10.1093/emboj/cdg233 PMC155991

[36] Wilkie GS, Zimyanin V, Kirby R, Korey C, Francis-Lang H, et al (2001) Small bristles, the *Drosophila* ortholog of NXF1, is essential for mRNA export throughout development. RNA 7: 1781–1792. 11780634 PMC1370217

[37] Katahira J (2012) mRNA export and the TREX complex. Biochimica et Biophysica Acta (BBA) 18119: 507–513. 22178508 10.1016/j.bbagrm.2011.12.001

[38] Manley J (2002) Nuclear coupling: RNA processing reaches back to transcription. Nature Structural Biology 9: 790–791. 12402032 10.1038/nsb1102-790

[39] Bregman A, Avraham-Kelbert M, Barkai O, Duek L, Guterman A, et al (2011) Promoter elements regulate cytoplasmic mRNA decay. Cell 147: 1473–1483. 22196725 10.1016/j.cell.2011.12.005

[40] Trcek T, Larson D, Moldón A, Query C, Singer R (2011) Single-molecule mRNA decay measurements reveal promoter-regulated mRNA stability in yeast. Cell 147: 1484–1497. 22196726 10.1016/j.cell.2011.11.051 PMC3286490

[41] Herold A, Klymenko T, Izaurralde E (2001) NXF1/p15 heterodimers are essential for mRNA nuclear export in *Drosophila* . RNA 7: 1768–1780. 11780633 PMC1370216

[42] Cullen B (2003) Nuclear mRNA export: insights from virology. Trends Biochem Sci 28: 419–424. 12932730 10.1016/S0968-0004(03)00142-7

[43] Grüter P, Tabernero C, von Kobbe C, Schmitt C, Saavedra C, et al (1998) TAP, the human homolog of Mex67p, mediates CTE-dependent RNA export from the nucleus. Mol Cel 1: 649–659. 10.1016/s1097-2765(00)80065-9 9660949

[44] Lei H, Dias A, Reed R (2011) Export and stability of naturally intronless mRNAs require specific coding region sequences and the TREX mRNA export complex. Proc Natl Acad Sci U S A 108: 17985–17990. 22010220 10.1073/pnas.1113076108 PMC3207654

[45] Le Hir H, Nott A, Moore M (2003) How introns influence and enhance eukaryotic gene expression. Trends Biochem Sci 28: 215–220. 12713906 10.1016/S0968-0004(03)00052-5

[46] Dai H, Yoshimatsu T, Long M (2006) Retrogene movement within- and between-chromosomes in the evolution of Drosophila genomes. Gene 385: 96–102. 17101240 10.1016/j.gene.2006.04.033

[47] Sorourian M, Betran E (2010) Turnover and lineage-specific broadening of the transcription start site in a testis-specific retrogene. Fly 4: 3–11. 20160503 10.4161/fly.4.1.11136 PMC2855778

[48] Bai Y, Casola C, Betran E (2008) Evolutionary origin of regulatory regions of retrogenes in Drosophila. BMC Genomics 9: 241. 18498650 10.1186/1471-2164-9-241 PMC2413143

[49] Fletcher J, Burtis K, Hogness D, Thummel C (1995) The *Drosophila E74* gene is required for metamorphosis and plays a role in the polytene chromosome puffing response to ecdysone. Development 121: 1455–1465. 7789275 10.1242/dev.121.5.1455

[50] White-Cooper H (2004) Spermatogenesis: analysis of meiosis and morphogenesis. In: Henderson D, editor. *Drosophila* Cytogenetics Protocols. Totowa, New Jersey: Humana Press. pp. 45–75. 10.1385/1-59259-665-7:45 14707342

[51] Morris C, Benson E, White-Cooper H (2009) Determination of gene expression patterns using in situ hybridization to *Drosophila* testes. Nature Protocols 4: 1807–1819. 20010932 10.1038/nprot.2009.192

[52] Thummel C, Boulet A, Lipshitz HD (1988) Vectors for *Drosophila* P-element-mediated transformation and tissue culture transfection. Gene 74: 445–456. 3246353 10.1016/0378-1119(88)90177-1

[53] Parker L, Gross S, Alphey L (2001) Vectors for the expression of tagged proteins in *Drosophila* . Biotechniques 31: 1280–1286. 11768656 10.2144/01316st01

[54] Dietzl G, Chen D, Schnorrer F, Su K, Barinova Y, et al (2007) A genome-wide transgenic RNAi library for conditional gene inactivation in *Drosophila* . Nature 448: 151–156. 17625558 10.1038/nature05954

[55] Tweedie S, Ashburner M, Falls K, Leyland P, McQuilton P, et al (2009) FlyBase: enhancing *Drosophila* Gene Ontology annotations. Nucleic Acids Research 37: D555–D559. 18948289 10.1093/nar/gkn788 PMC2686450

