# Supplementary figures and images for: The RNA Export Factor, Nxt1, Is Required for Tissue Specific Transcriptional Regulation
Source: PLoS Genet. 2013 Jun 6;9(6):e1003526. doi: 10.1371/journal.pgen.1003526 (PMC3674997; doi:10.1371/journal.pgen.1003526)

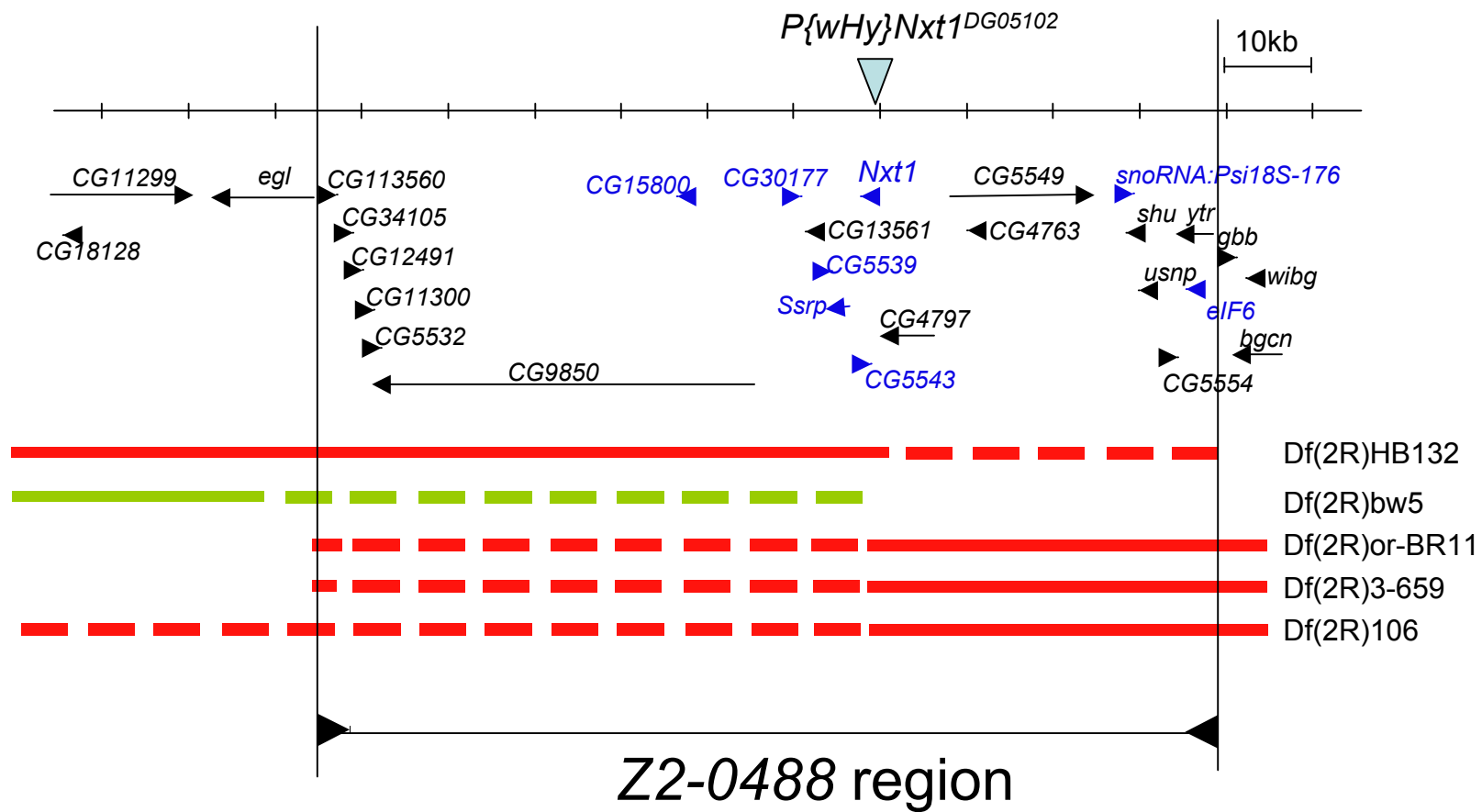

Supplement: Figure S1 — Cloning of Z2-0488. The meiotic arrest phenotype of Z2-0488 was mapped by recombination to a region of chromosome 2R. From published data [55] we defined the proximal end of the Z2-0488 region by the distal breakpoint of Df(2R)bw5, which uncovers egl, but does not uncover Z2-0488; and by the proximal breakpoint of Df(2R)or-BR11, which uncovers Z2-0488 but not egl. The distal end of the Z2-0488 region was defined by the proximal breakpoint of Df(2R)HB132, which uncovers egl and Z2-0488, but does not uncover gbb. Thus Z2-0488 lies between egl and gbb. The lethality of P{wHy}Nxt1DG05102 had the same complementation pattern with respect to deficiencies as Z2-0488. Candidate genes from the region were sequenced (blue), and the only mutation detected in Z2-0488 was in Nxt1. (PDF) [file pgen.1003526.s001.pdf]

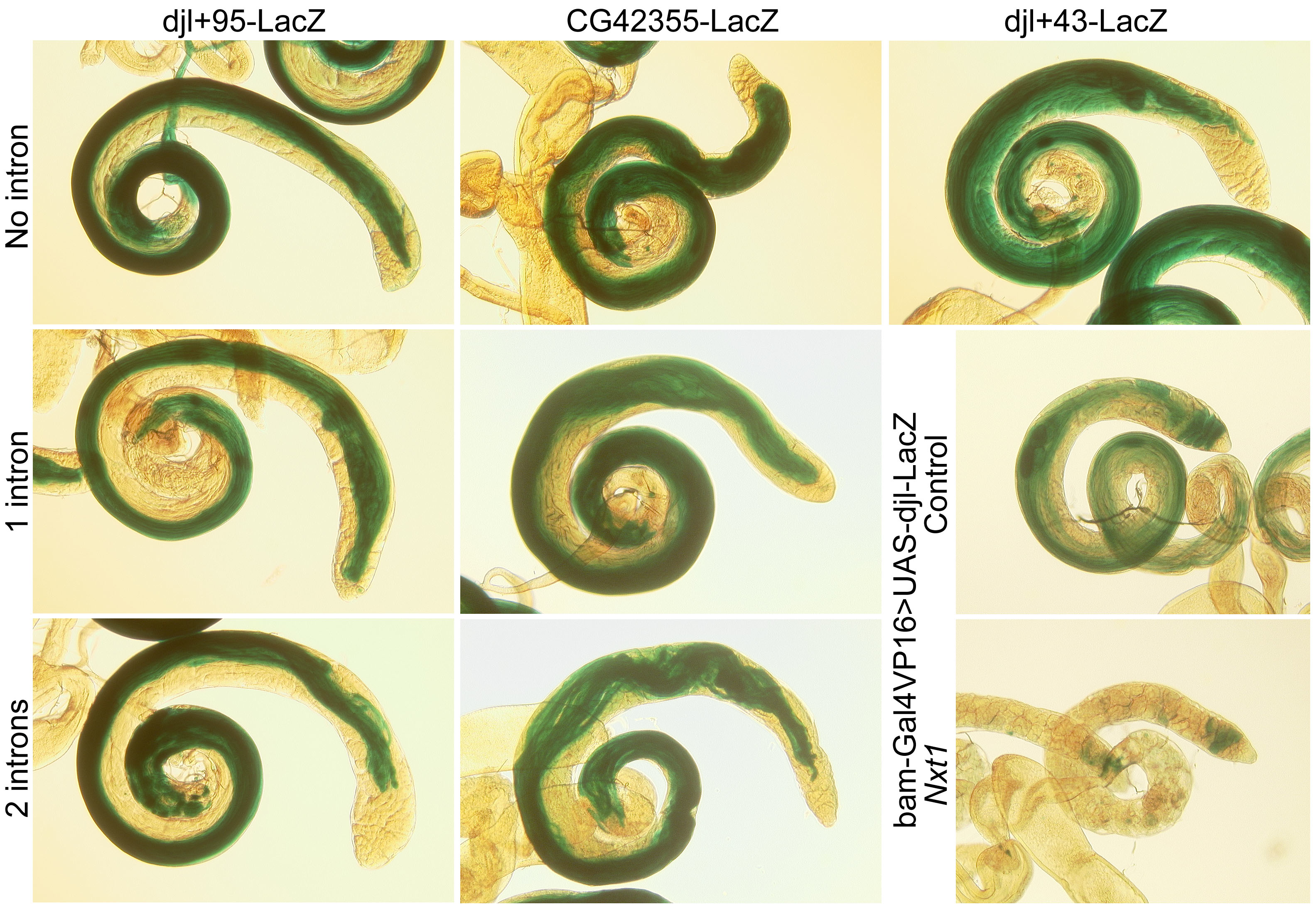

Supplement: Figure S3 — Reporter construct LacZ protein expression patterns. Beta-galactosidase activity staining reveals that translation of both djl+95-LacZ and 42355-LacZ is delayed until spermatid differentiation, while djl+43-LacZ translation is detected in late primary spermatocytes. Insertions of introns into the reporters does not alter the translation timing or protein expression pattern. Expression of the djl+95-LacZ transcript with the UAS construct results in earlier expression than of the endogenous transcript. There is a bi-phasic protein expression profile, with translation in early primary spermatocytes, repression in later primary spermatocytes and early spermatids, and a second wave of translation in spermatids. When expressed in Nxt1Z2-0488/Nxt1DG05102 testes the protein is expressed in early primary spermatocytes, and repressed in the later primary spermatocytes. (TIFF) [file pgen.1003526.s003.tif]

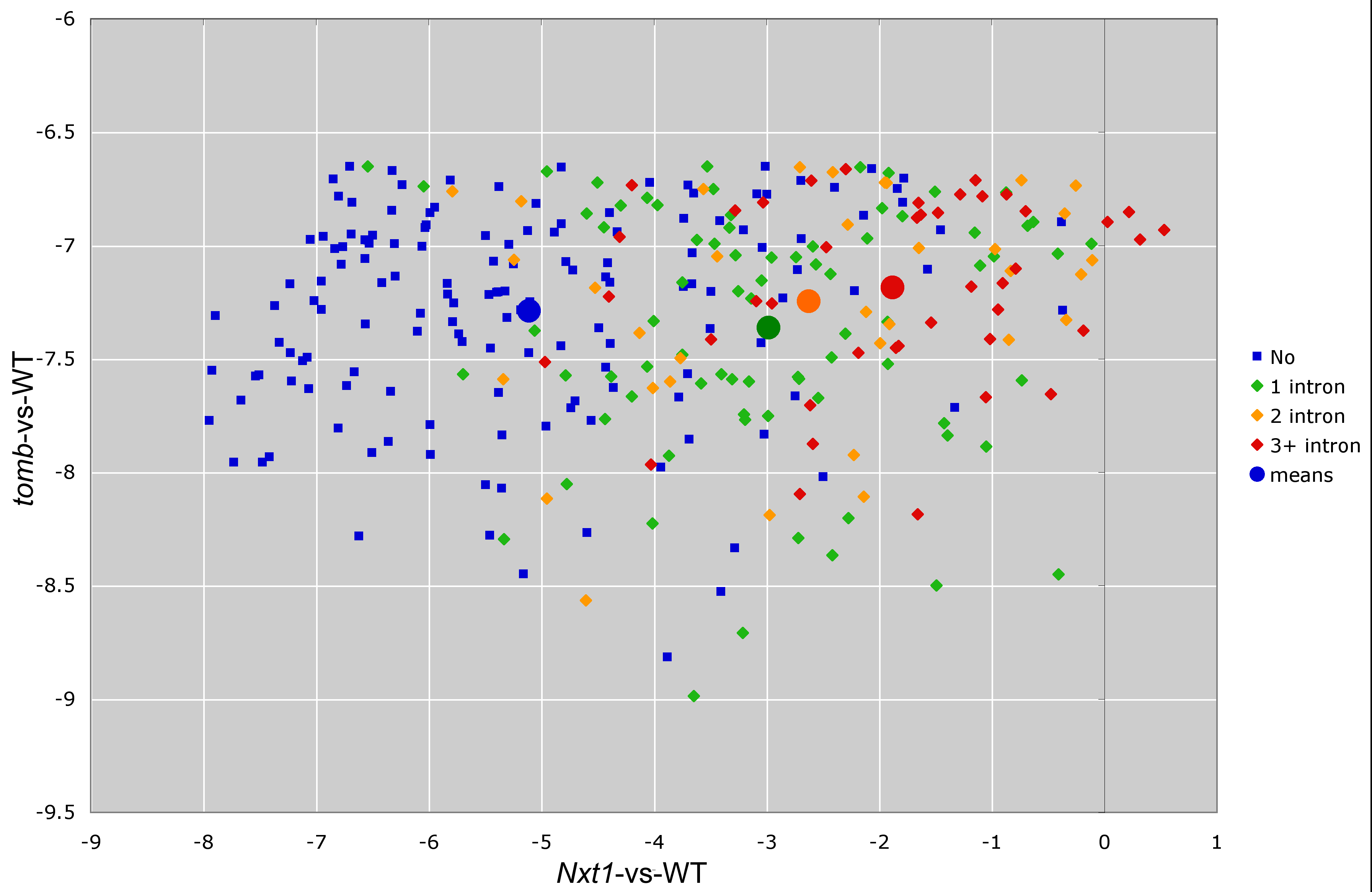

Supplement: Figure S4 — Effect of intron number on TMAC-dependent gene expression in Nxt1Z2-0488/Nxt1DG05102 testes. The scatter plot shows the 300 transcripts most down-regulated in tomb mutant testes. The array data of log2 transformed relative expression of transcripts in TMAC mutant testes (tomb) compared to wild type is plotted on the y-axis, while the relative expression in Nxt1Z2-0488/Nxt1DG05102 testes compared to control is on the x-axis. Transcripts from genes with no introns are indicated by blue squares, while those from genes with 1, 2 or 3 or more are indicated with green, orange and red diamonds respectively. Mean expression fold changes are show with large dots. (TIF) [file pgen.1003526.s004.tif]

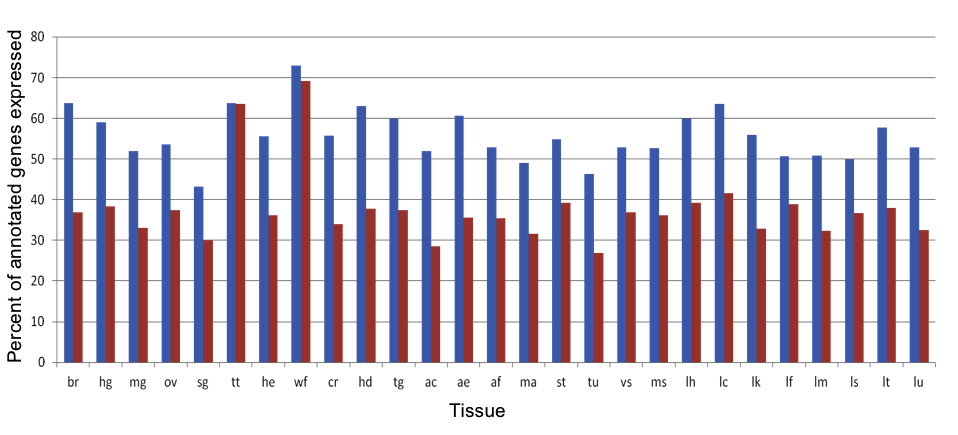

Supplement: Figure S5 — Expression of genes with and without introns in adult and larval fly tissues. The proportion of all annotated genes with (blue) or without introns (red) whose expression is detected in a specific tissue. Only testis expresses a the same proportion of intron-less genes as intron containing genes. br = brain; hg = hindgut; mg = midgut; sg = salivary gland; tt = testis; he = heart; wf = whole fly; cr = crop; hd = head; tg = thoracio-abdominal ganglion; ac = adult carcass; ae = eye; af = adult fatbody; ma = male accessory gland; st = S2 cells; tu = malpigian tubule; vs. = virgin female spermatheca; ms = mated female spermatheca; lh = larval hindgut; lc = larval CNS; lk = larval carcass; lm = larval midgut; ls = larval salivary gland; lt = larval trachea; lu = larval malpigian tubule. (TIF) [file pgen.1003526.s005.tif]
