# Supplementary material for: The RNA Export Factor, Nxt1, Is Required for Tissue Specific Transcriptional Regulation
Source: PLoS Genet. 2013 Jun 6;9(6):e1003526. doi: 10.1371/journal.pgen.1003526 (PMC3674997; doi:10.1371/journal.pgen.1003526)
Supplement: Figure S2 — Nxt1-dependent transcripts are also highly aly-dependent but not vice-versa. Scatter plots of microarray data comparing gene expression in wild type with Nxt1 mutant (A, C, E) or with aly mutant (B, D, F). Probes that pass the threshold filters (16×, 8× or 4× down in mutants vs. wild type) are coloured (A, B). (C, D) expression plots as in A or B are recoloured according to how the probes behave in the other mutant genotype. Probes in purple in panel D (wt vs. aly) are those whose expression is 16 fold or more reduced in Nxt1 mutants compared to wild type. These all cluster in the bottom right hand corner of the plot, ie they have high expression in wild type and low expression in aly. Probes in red in panel C (wt vs. Nxt1) are those whose expression is 16 fold or more reduced in aly mutants compared to wild type. These spread up the right hand side of the plot, ie they have high expression in wild type, and high, medium or low expression in Nxt1 mutants. (E, F) The same plots as C and D except that the probes that did not pass the filters (ie less than 4× change), coloured white, are plotted on top of the other data rather than behind. Very few white dots are present in the bottom right corner in panel E while many white dots are in this region in F. This demonstrates that virtually all genes that depend on Nxt1 also depend on aly, but that not all genes that depend on aly also depend on Nxt1. The expression of known meiotic arrest genes in mutant testes, is shown by large dots in panels A and B. None of the known meiotic arrest genes have reduced expression in Nxt1 or aly testes. (PDF) [file pgen.1003526.s002.pdf]

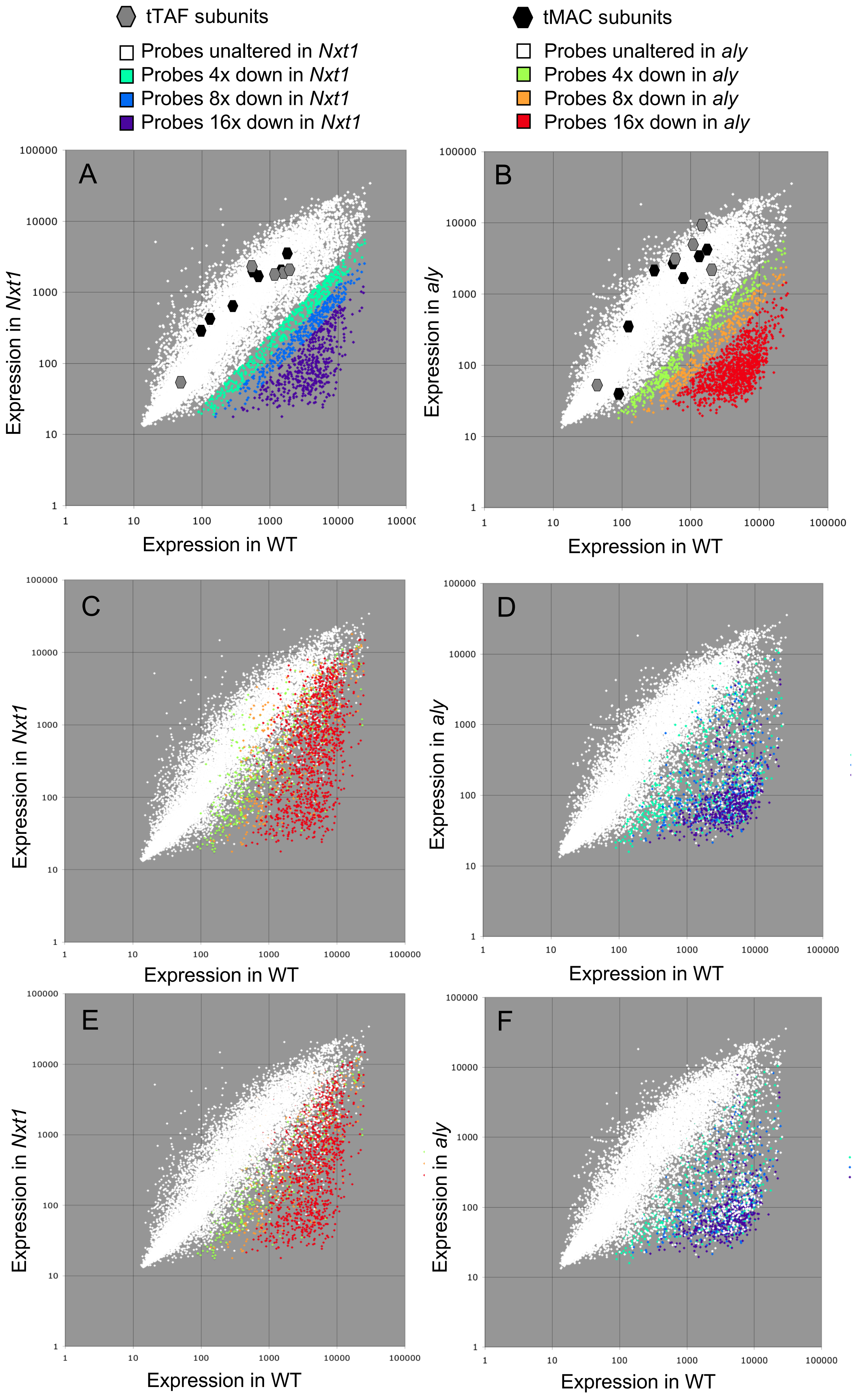

tTAF subunits
Probes unaltered in *Nxt1*
Probes 4x down in *Nxt1*
Probes 8x down in *Nxt1*
Probes 16x down in *Nxt1*
tMAC subunits
Probes unaltered in *aly*
Probes 4x down in *aly*
Probes 8x down in *aly*
Probes 16x down in *aly*
A
B
C
D
E
F
Expression in *Nxt1*
Expression in *aly*
Expression in WT
1
10
100
1000
10000
100000
