# Supplementary material for: The RNA Export Factor, Nxt1, Is Required for Tissue Specific Transcriptional Regulation
Source: PLoS Genet. 2013 Jun 6;9(6):e1003526. doi: 10.1371/journal.pgen.1003526 (PMC3674997; doi:10.1371/journal.pgen.1003526)
Supplement: Text S1 — This file contains details of the cloning of introns into the reporter constructs, including the sequences used in their native context and in the reporter construct context. This file also contains sequences for all the PCR primers used in the manuscript. (DOC) [file pgen.1003526.s006.doc]

## Supplementary Materials and Methods.

### Plasmid construction

The *djl* intron and the *CG42355* introns, inserted at sites that closely matched the endogenous flanking sequence, were synthesised (MWG Eurofins) within a 379bp fragment from the 5' end of the reporter gene, flanked by Kpn1 and Bsu36I restriction sites for subcloning. These fragments then replaced the intron-less fragment in pCaSpeR4-AUG-betaGAL djl-lacZ -555/+95 and CG42355-LacZ -177/+136.

Purple indicates Kpn1 restriction site (in polylinker). ATG is *LacZ* start codon. Intron is in cyan. Underlined are flanking nucleotides that match the endogenous flanking sequence for the introns being inserted. Green is Bsu36I restriction site in LacZ.

>djl-intron in endogenous location

TTAAGAAAAAAAGgtaagagctcaaacggtgacctcttaaatagataacttatacttgttcgtactagAAATTCTCAA

>djl-intron inserted into Kpn1-Bsu36I fragment of pCaSpeR4-AUG-betaGal

ggtaccgctagagtcgaccaattcccggggatcgaaagagcctgctaaagcaaaaaaggtaagagctcaaacggtgacctcttaaatagataacttatacttgttcgtactagaagtcaccATGtcgtttactttgaccaacaagaacgtgattttcgttgccggtctgggaggcattggtctggacaccagcaaggagctgctcaagcgcgatcccgtcgttttacaacgtcgtgagtgggaaaaccctggcgttacccaacttaatcgccttgcagcacatccccctttcgccagctggcgtaatagcgaagaggcccgcaccgatcgcccttcccaacagttgcgcagcctgaatggcgaatggcgctttgcctggtttccggcaccagaagcggtgccggaaagctggctggagtgcgatcttcctgagg

>CG42355-intron1 in endogenous location

GTATTTTGAGgtaagtatgagccaattgtgcttctacgccacagtatttacaacgccggttgaccagGTGAGACCTAA

>CG42355-intron2 in endogenous location

TCGCACGGCGgtaagtatatgcctaaatttccaaaaccctgattctaaaccaaaatgttttgaagGGAGGGAGGG

>CG42355-introns inserted into Kpn1-Bsu36I fragment of pCaSpeR4-AUG-betaGal

ggtaccgctagagtcgaccaattcccggggatcgaaagagcctgctaaagcaaaaaagaagtcaccATGtcgtttactttgaccaacaagaacgtgattttcgttgccggtctgggaggtaagtatgagccaattgtgcttctacgccacagtatttacaacgccggttgaccaggcattggtctggacaccagcaaggagctgctcaagcgcgatcccgtcgttttacaacgtcgtgagtgggaaaaccctggcgttacccaacttaatcgccttgcagcacatccccctttcgccagctggcgtaatagcgaagaggcccgcaccgatcgcccttcccaacagttgcgcagcctgaatggcgaatggcgctttgcctggtttccggcaccagaagcggtaagtatatgcctaaatttccaaaaccctgattctaaaccaaaatgttttgaaggtgccggaaagctggctggagtgcgatcttcctgagg

An UAS-hsp70 synthetic fragment was synthesised as below. This was cloned into the EcoRI and BamHI sites of the pCaSpeR4-AUG-betaGal polylinker. EcoR1 5x UAS hsp70 minimal promoter to -1 djl 5'UTR (+1 to +95) BamHI.

>UAS-hsp70-djl95

GAATTCGGTCGGAGTACTGTCCTCCGAGCGGAGTACTGTCCTCCGAGCGGAGTACTGTCCTCCGAGCGGAGTACTGTCCTCCGAGCGGAGTACTGTCCTCCGAGCGGAGACTCTAGCGAGCGCCGGAGTATAAATAGAGGCGCTTCGTCTACGGAGCGACAATTCAATTCAAACAAGAGTGTTCAAAAATTGTGAAGTTTGGTTATTTCCTTTGCTTAGCTTTTCGTTAAAAATTCATTTTCCACAAGAAAAATTCTAAATTTACCGTTATAGGATCC

### Primer sequences

| Name | Sequence (5'-3') | Application |
| --- | --- | --- |
| CG3927-F | CTGTGGACCGTGTCTCTCC | mRNA normalisation for Q-RT-PCR |
| CG3927-R | CCTAGGATCTTGCCAGTGAA | mRNA normalisation for Q-RT-PCR |
| LacZ-F3 | TGGCAGGGTGAAACGCAGGTCG | mRNA expression analysis Q-RT-PCR |
| LacZ-R3 | CCGTTCAGCAGCAGCAGACCAT | mRNA expression analysis Q-RT-PCR |
| djl-RT-F | TCGGTGCCTTCAGACTTCTG | mRNA expression analysis Q-RT-PCR |
| djl-RT-R | ATCACTCCAATGGAGCCGTC | mRNA expression analysis Q-RT-PCR |
| CG42355-RTF | TGCCGACCTAGACGAAGACGAG | mRNA expression analysis Q-RT-PCR |
| CG42355-RTR | CTTTCTTCTTCTGGTTCTTAGG | mRNA expression analysis Q-RT-PCR |
| ran-like-RTF | TTGGTGAGAGTATGCGGCAA | mRNA expression analysis Q-RT-PCR |
| ran-like-RTR | AGACGAATGGCTTCTCCACG | mRNA expression analysis Q-RT-PCR |
| CG11249-b | TAAACTCCTTATACACGCCACG | Nascent vs mature RNA |
| CG11249-c | GAGGTATATAGGGGGTCTCAG | Nascent vs mature RNA |
| CG11249-d | TGAAACACTGATCTCCGCCTC | Nascent vs mature RNA |
| CG11249-sf | ACACCATCTATTGGGCGGAGTC | Nascent vs mature RNA |
| Pif2-a | ACACACAAATCAAATTCAAGCCC | Nascent vs mature RNA |
| Pif2-c | CTGTTAGTTAGAGTACGGATGA | Nascent vs mature RNA |
| Pif2-d | AGTCATTGCAGCACGGTGGACA | Nascent vs mature RNA |
| Pif2-sf | CGGGTTCAAGCACGAATCGAA | Nascent vs mature RNA |
| CG4907-a | AAACTACACCCAACGTGGCA | Nascent vs mature RNA |
| CG4907-c | GGGATTCATGGGTAATTGGT | Nascent vs mature RNA |
| CG4907-sr | ACCGTGCAGACTTATCCCAT | Nascent vs mature RNA |
| CG17380-b | GGAGTCCTACGTAGTAAATC | Nascent vs mature RNA |
| CG17380-sf | GGCACTCAGACATGAGAGAT | Nascent vs mature RNA |
| CG17380-d | ATTCGGGTTTCCGCCACAA | Nascent vs mature RNA |
| CG16736-a | GTCCTGGGTATAACCGGATT | Nascent vs mature RNA |
| CG16736-c | ACCGGAAAATGCCTATTTATGT | Nascent vs mature RNA |
| CG16736-sr | GAAATTTCGATAGTTGCGCCT | Nascent vs mature RNA |
| CG32487-a | CCGTACATGTGCATCGACTT | Nascent vs mature RNA |
| CG32487-c | CTGTGAAGTGGCAATCCAAA | Nascent vs mature RNA |
| CG32487-sr | ATCTGTGCACATGGTGTCTC | Nascent vs mature RNA |
| CG10478-a | ACGATAGGAAACCTGGTGGA | Nascent vs mature RNA |
| CG10478-c | GGAATAATAGCAATAGCCTAG | Nascent vs mature RNA |
| CG10478-sr | GCCAGCCTTCGCAATTAAAT | Nascent vs mature RNA |
| CG14546-a1 | ATCAGGAGGCGATGCTCAAT | Nascent vs mature RNA |
| CG14546-a2 | GTCCAAGAGCTTTGAGCAAT | Nascent vs mature RNA |
| CG14546-c | GGACATCGGTGAAGTTGTGA | Nascent vs mature RNA |
| CG14546-sr | TGAGCGGCAATTTTGTTGGT | Nascent vs mature RNA |
| CG33125-a | CGATGAATGGAATCGGACAT | Nascent vs mature RNA |
| CG33125-c | GGACGATTGCCAAATGTCGT | Nascent vs mature RNA |
| CG33125-sr | CCGGGAGCTCGTCGCTTT | Nascent vs mature RNA |
| CG12699-b | TCCAACAGGCAAGTAAAAACGA | Nascent vs mature RNA |
| CG12699-c | TTGGACTGCAAGAAAAAGAGAAG | Nascent vs mature RNA |
| CG12699-d | TATCAGCATCGGATGTAGTGG | Nascent vs mature RNA |
| CG12699-sf | CCTCCAACAGTCCAAAATGTCA | Nascent vs mature RNA |
| ocn-b | GCTGCAAGGTAAGTGGATGAAG | Nascent vs mature RNA |
| ocn-c | CAAAAGTCTGCAGGCAGTCTC | Nascent vs mature RNA |
| ocn-d | CAGGTTAAAGGTCCAAGACAGC | Nascent vs mature RNA |
| ocn-sf | GTGCTGCAAGACTTTTGGGGA | Nascent vs mature RNA |
| CG42355-F | GCTCTAGAATTCAAAATAATAAGGATGACTC | Plasmid construction |
| CG42355-R | GCTCTAGGATCCGATAAAGTGCACTTTTAATAC | Plasmid construction |
| Nxt1-F | ATGGACAGCGATTTGAAAGCCAA | Plasmid construction |
| Nxt1-R | ATCTCAGACCTCCTGCATTCGGTA | Plasmid construction |
| LacZ-spl-F | TCGAAAGAGCCTGCTAAAGC | PCR to confirm splicing |
| LacZ-spl-R | CGCACTCCAGCCAGCTTTC | PCR to confirm splicing |
| 5’lacZ | CAACCCGTGGTCGGCTTACG | RNA probe synthesis |
| 3’lacZ-T3 | GCAACGAATTAACCCTCACTAAAGGGCGTTAGGGTCAATGCGGG | RNA probe synthesis |
| 5’CG11249-a | CACGCGGACCACCTCAAGGG | RNA probe synthesis |
| 3’CG11249-a-T3 | GCAACGAATTAACCCTCACTAAAGGGTACCACGGAACGCGCCAAGC | RNA probe synthesis |
| 5’CG11249-b | ACCGTGGAAGCGAGATGGCG | RNA probe synthesis |
| 3’CG11249-b-T3 | GCAACGAATTAACCCTCACTAAAGGGTGCCCTCCTGGCGGAACTGA | RNA probe synthesis |
| 5'CG15177 | CAAGTTCTCGGCCCTGTACG | RNA probe synthesis |
| 3'CG15177-T3 | GCAACGAATTAACCCTCACTAAAGGGTTGTTGTGAAACAACCG | RNA probe synthesis |
| 5'ran-like-SP6 | GGAAGCGGCCGCATGCAACCTCAAGAGGAAGTGAA | RNA probe synthesis |
| 3'ran-like-T3 | GGAAGGTACCATTGAATAAGCTTTCCACTTGAC | RNA probe synthesis |
| 5’djl | CTCCCCAATCCCATGCACCG | RNA probe synthesis |
| 3’djl | GCATTGCTCCCTCTCCGCAA | RNA probe synthesis |
| 5’CG42355-a | CGCCATGGGAGCATCGACAG | RNA probe synthesis |
| 3’CG42355-a | CGCGGTGCTCATTGCAGGAT | RNA probe synthesis |
| 5’CG42355-b | GGTCAGCAGAAAATGCCTGGGG | RNA probe synthesis |
| 3’CG42355-b | TCCTCCTTTGGTACTCGCGCA | RNA probe synthesis |
| 5'CG18628-SP6 | ATTTAGGTGACACTATAGAATGAAGTTCCTACTCGTGTGC | RNA probe synthesis |
| 3'CG18628-T3 | TAACCCTCACTAAAGGGCGGTTTAAGATCGATGAAG | RNA probe synthesis |
